# Interactive parallel sex pheromone circuits that promote and suppress courtship behaviors in the cockroach

**DOI:** 10.1093/pnasnexus/pgae162

**Published:** 2024-04-15

**Authors:** Kosuke Tateishi, Takayuki Watanabe, Mana Domae, Atsushi Ugajin, Hiroshi Nishino, Hiroyuki Nakagawa, Makoto Mizunami, Hidehiro Watanabe

**Affiliations:** Department of Earth System Science, Faculty of Science, Fukuoka University, 8-19-1 Nanakuma, Jonan-ku, Fukuoka 814-0180, Fukuoka, Japan; School of Biological and Environmental Sciences, Kwansei Gakuin University, 1 Gakuen Uegahara, Sanda 669-1330, Hyogo, Japan; Research Center for Integrative Evolutionary Science, The Graduate University for Advanced Studies, Shonan Village, Hayama 240-0193, Kanagawa, Japan; Research Institute for Electronic Science, Hokkaido University, Kita 12, Nishi 6, Kita-ku, Sapporo 060-0812, Hokkaido, Japan; Laboratory Sector, JT Biohistory Research Hall, 1-1 Murasaki-cho, Takatsuki 569-1125, Osaka, Japan; Research Institute for Electronic Science, Hokkaido University, Kita 12, Nishi 6, Kita-ku, Sapporo 060-0812, Hokkaido, Japan; Department of Earth System Science, Faculty of Science, Fukuoka University, 8-19-1 Nanakuma, Jonan-ku, Fukuoka 814-0180, Fukuoka, Japan; Research Institute for Electronic Science, Hokkaido University, Kita 12, Nishi 6, Kita-ku, Sapporo 060-0812, Hokkaido, Japan; Department of Earth System Science, Faculty of Science, Fukuoka University, 8-19-1 Nanakuma, Jonan-ku, Fukuoka 814-0180, Fukuoka, Japan

**Keywords:** sex pheromone, olfactory receptor, pheromone processing, courtship behavior, cockroach

## Abstract

Many animals use multicomponent sex pheromones for mating, but the specific function and neural processing of each pheromone component remain unclear. The cockroach *Periplaneta americana* is a model for studying sex pheromone communication, and an adult female emits major and minor sex pheromone components, periplanone-B and -A (PB and PA), respectively. Attraction and courtship behaviors (wing-raising and abdominal extension) are strongly expressed when adult males are exposed to PB but weakly expressed when they are exposed to PA. When major PB is presented together with minor PA, behaviors elicited by PB were impaired, indicating that PA can both promote and suppress courtship behaviors depending on the pheromonal context. In this study, we identified the receptor genes for PA and PB and investigated the effects of knocking down each receptor gene on the activities of PA- and PB-responsive sensory neurons (PA- and PB-SNs), and their postsynaptic interneurons, and as well as effects on courtship behaviors in males. We found that PB strongly and PA weakly activate PB-SNs and their postsynaptic neurons, and activation of the PB-processing pathway is critical for the expression of courtship behaviors. PA also activates PA-SNs and the PA-processing pathway. When PA and PB are simultaneously presented, the PB-processing pathway undergoes inhibitory control by the PA-processing pathway, which weakens the expression of courtship behaviors. Our data indicate that physiological interactions between the PA- and PB-processing pathways positively and negatively mediate the attraction and courtship behaviors elicited by sex pheromones.

**Significance Statement**

To locate and copulate mates, insects utilize sex pheromones composed of major and minor components, but the specific functions and neural processing of each component remain unclear. Here, we successfully identified receptor genes for major and minor sex pheromone components in the American cockroach. By knocking down these receptors, we investigated the physiological and behavioral roles of major and minor sex pheromone components in the cockroach. We showed that physiological interactions between major and minor sex pheromone processing pathways play critical roles in controlling courtship behaviors. These interactions allow the minor component to positively or negatively modulate courtship behaviors elicited by the major component, depending on the pheromonal context.

## Introduction

Pheromones are semiochemicals that effectively trigger species-specific behaviors in conspecifics, such as aggregation, courtship, and social behaviors. In many insect species, sex pheromones emitted by adult females consist of a major component and several minor components, and they collectively attract and elicit typical courtship behaviors in adult males (1–5). Generally, attraction and courtship behaviors are elicited by the major sex pheromone component alone, and the minor components modulate the behavioral activity elicited by the major component (1, 6). In lepidopterans, the neural mechanisms underlying the attractive functions of the major component have been deciphered, but those achieving the modulative functions of the minor sex pheromone components remain poorly understood (7–11).

The American cockroach *Periplaneta americana* is a common and widespread pest in urban environments, and the sex pheromones and courtship behaviors they elicit have been extensively investigated to control populations of pest insects (5, 12). In *P. americana*, an adult female emits two sex pheromone components: the major component periplanone-B (PB) and the minor component periplanone-A (PA) (13). In response to the sex pheromones, the sexually aroused adult male moves towards to the female with wing-fluttering (partial wing-raising). When the male antennates the female, the male raises its wings (full wing-raising) and extends its abdomen to release an attractant pheromone that directs the female to his dorsum (5). A series of attraction and subsequent male courtship behaviors can be induced by either PB or PA when they are presented alone. However, PA was approximately 30–50 times less effective in terms of behavioral threshold concentrations (14–16). When PA is presented together with PB, it negatively modulates the attraction triggered by PB in a dose-dependent manner (14, 17). These results suggest that the minor sex pheromone component PA, has both promotive and suppressive functions in courtship behaviors depending on the pheromonal context. Therefore, it is reported in the cockroach that PB is used for long-distance attraction, whereas PA is used as an arrestant and influences male behavior closer to the female (5, 17).

In *P. americana*, the PA- and PB-processing pathways have been characterized at the cellular level from the peripheral to higher brain centers. PA and PB are respectively detected by two different sensory neurons, PA-SNs and PB-SNs, in the adult male-antennae-specific *single walled-*B (*sw-*B) sensilla (18–21). A *sw*-B sensillum houses one PA-SN and one PB-SN in addition to two olfactory sensory neurons (OSNs), which respond to various general odors (18–21). The axons of PA-SNs and PB-SNs selectively project to two macroglomeruli, the A- and B-glomeruli, where they synapse onto A-projection neurons (A-PNs) and B-PNs, respectively (20, 22). The axons of both A-PNs and B-PNs run through the medial antennal lobe tract, and project to the ipsilateral mushroom body (MB) and lateral horn (LH) (23–25). The axon terminals of A-PNs and B-PNs synapse onto distinct sets of Kenyon cells (KCs) in the MB calyces, and they converge into the antero-medial region of the LH (am-LH) (24–26). Therefore, the cockroach is equipped with anatomically segregated parallel PA- and PB-processing pathways from the peripheral to higher brain centers.

To investigate how the functional differences between PA and PB are achieved by the parallel PA- and PB-processing pathways, we identified functional PA and PB receptors in the cockroach, and conducted an RNAi-mediated knockdown study of the identified receptors. In insects, odorant receptors (ORs) form heteromeric ligand-gated cation channels composed of a ligand-specific tuning receptor (ORx) and a co-receptor (ORco) (27). Recently, we demonstrated that *P. americana* ORco (*Pame*ORco) is essential in PA and PB receptions and courtship behaviors elicited by sex pheromones, suggesting that male adult cockroaches possess ORxs tuned to PA and PB (19). It has been reported in the cockroach antennae that four *OR*s, *PameOR1, PameOR2, PameOR53*, and *PameOR62*, exhibit male-biased expression in adult antennae (28). Here, we hypothesized that these four ORs include sex pheromone ORs, which form complexes with *Pame*ORco in PA-SNs or PB-SNs. By combining systemic RNAi targeting of the putative sex pheromone *PameORs* and electrophysiological recordings from PA-SNs and PB-SNs, we successfully identified the functional PA and PB receptors for the first time. Knockdown of PA or PB receptor genes revealed that PA-SNs were selectively tuned to PA, but PB-SNs were broadly tuned to both PB and PA. In addition, our investigation of sex pheromone responses of B-PNs revealed that physiological interactions from the PA-processing pathway to the PB-processing pathways in the brain. The interactive parallel sex pheromone circuits play critical roles in promotive and suppressive control of courtship behaviors triggered by major PB and minor PA in American cockroaches.

## Results

### Identification of PA and PB receptors

**Fig. 1.**
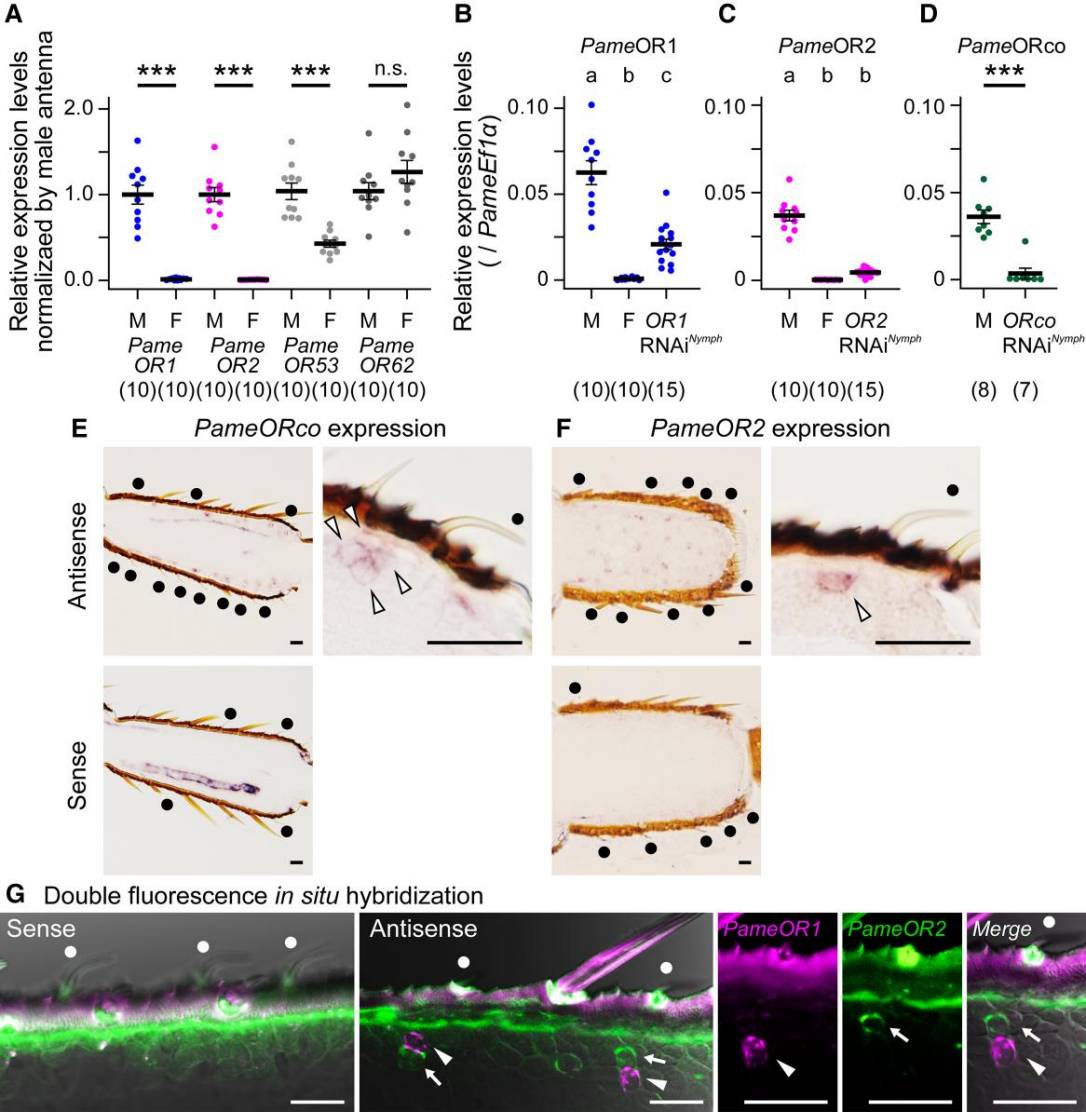
Expression patterns and RNA interference efficacies of *PameORs*. (A) Sex-biased expressions of *PameORs* in the adult antennae. Expression levels of four putative sex pheromone-responsive *PameORs* are examined in the antennae of 11-day-old adult males and females. B–D) RNAi efficiencies of *PameOR1* (B), *PameOR2* (C), and *PameORco* (D) dsRNA injection. Expression levels of *PameOR*s are examined in 11-day-old adult males, females and RNAi males. To prepare RNAi cockroaches, 4 μg of dsRNA of the target gene was injected into 7-day-old last-instar nymphs (RNAi*^Nymph^*). Expression levels of *PameOR*s were normalized with that of *PameEf1α*. The number of samples is indicated in parentheses. The different letters above each plot indicate significant differences (ANOVA with post hoc Tukey–Kramer test; *p* < 0.05). Expression levels of *PameORco* were obtained from Tateishi et al. (19). E, F) Expressions of *PameORco* (E) or *PameOR2* (F) in the antennae of adult male cockroaches. In single *sw*-B sensilla, the *PameORco* anti-sense probe labeled four OSNs (arrowheads in E), whereas the *PameOR2* anti-sense probe selectively labeled one of four OSNs in a single *sw*-B sensilla (arrowhead in F). Sense RNA probes did not label any OSNs under the same conditions. G) Visualization of *PameOR1* (magenta), *PameOR2* (green) expressions in the antennae of adult male cockroaches. The *PameOR1* and *PameOR2* expressions are simultaneously detected in antennae of male adult cockroaches by double-colored fluorescence *in situ* hybridization. DIG- and FLU-labeled probes were synthesized for *PameOR1* and *PameOR2*, respectively. In single *sw*-B sensilla, an OSN expressing *PameOR1* (arrowheads) paired with an OSN expressing *PameOR2* (arrows). Scale bars in E–G = 20 μm. *Sw*-B sensilla are pointed by black dots in E and F, and by white dots in G.

**Fig. 2.**
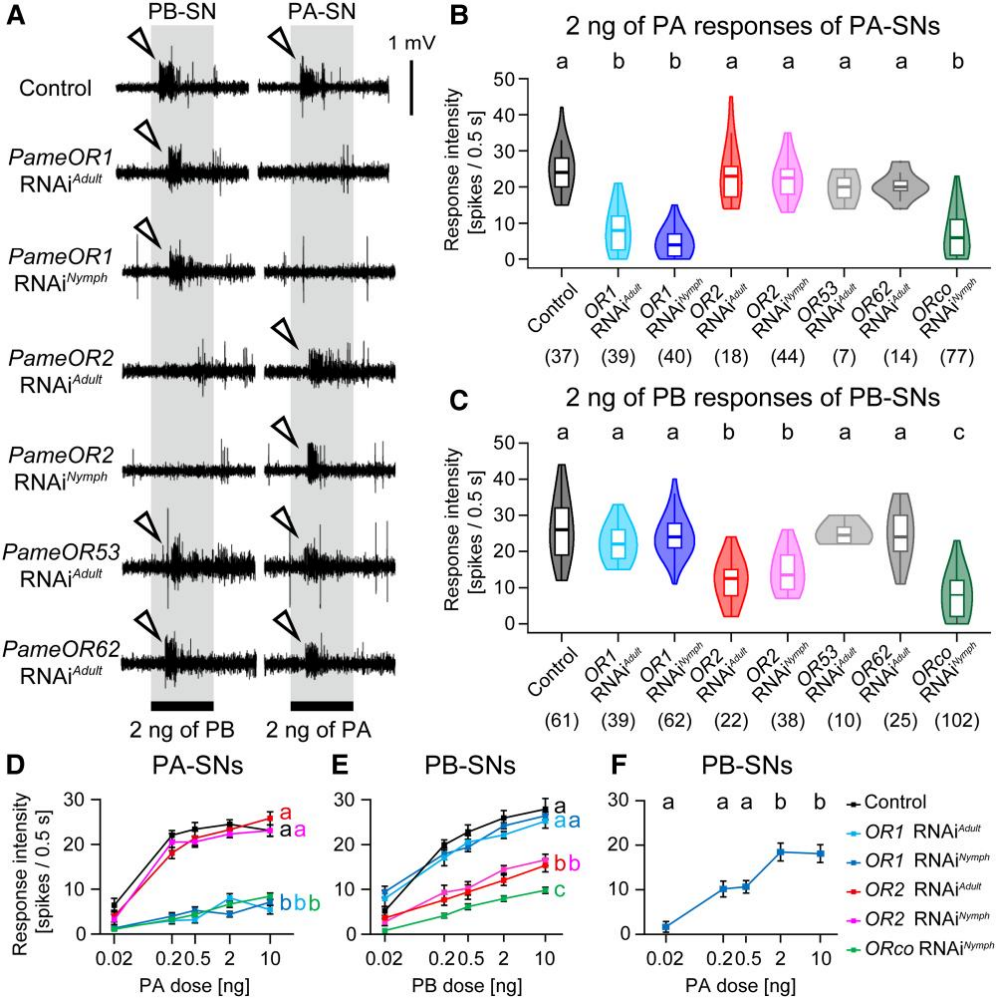
Functional characterizations of PA and PB receptors. A) Typical sex pheromone responses of single *sw*-B sensilla from putative sex pheromone receptor RNAi cockroaches. We injected the target *PameOR* dsRNA into last-instar nymph (RNAi*^Nymph^*) or 4-day-old adult (RNAi*^Adult^*) male cockroaches and performed single sensillum recordings using 11-day-old adult male cockroaches. PB-SNs and PA-SNs exhibit phasic responses to PB and PA, respectively (arrowheads), defined by the cross-adaptation odor stimuli system (Fig. S2). B, C) PA responses of PA-SNs (B) and PB responses of PB-SNs (C) in *PameORs* RNAi cockroaches. In each violin plot shown in this and following figures, the median is indicated by the internal line, the 25th to 75th percentiles are indicated by the white box, and the whiskers indicate 1.5× the interquartile range. Different letters above columns indicate significant differences (ANOVA with post hoc Tukey–Kramer test; *p* < 0.05). D, E) Dose response curves of PA-SNs to PA (D) and PB-SNs to PB (E) in each RNAi group. Response intensities of PA-SNs and PB-SNs to each of sex pheromone concentrations are shown in Fig. S3. F) Dose responses of PB-SNs to PA revealed by *PameOR1* RNAi cockroaches. Response intensities are denoted as means ± SEM. Different letters in each of panels indicate significant differences (one-way or two-way ANOVA with post hoc Tukey–Kramer test; *p* < 0.05). Number of recorded sensory neurons used in each plot is shown in parentheses and Table S2. Individual *P*-values calculated by statistical analyses are also shown in Table S2. Responses to sex pheromones in control (no-treatment and *β-lac* RNAi) and *PameORco* RNAi*^Nymph^* cockroaches were obtained from Tateishi et al. (19).

We obtained full-length cDNA clones of four OR genes (Fig. S1 and Table S1; *PameOR1*; GenBank ID: LC781791, *PameOR2*; LC781792, *PameOR53*; LC781793, and *PameOR62*; LC781794) which have been reported to have male-biased expression in the antennae (28). We re-analyzed expression levels of the four ORs in antennae between sexes and found that the expression levels of *PameOR1, PameOR2*, and *PameOR53* were significantly higher in adult males than in adult females (Fig. 1A and Table S1). To determine their roles in sex pheromone reception, we performed RNAi-based functional analysis. We injected dsRNA targeting each *PameOR* into 7-day-old last-instar nymphs (RNAi*^Nymph^*) or 4-day-old adults (RNAi*^Adult^*), and assessed RNAi efficiencies in 11-day-old adult males by RT-qPCR (Figs. 1B–D) and single sensillum recordings (SSRs) from the *sw*-B sensilla (Figs. 2, S2–4). Because sex pheromone responses of PB-SNs and PA-SNs were not different between no-treatment cockroaches and *Escherichia coli β-lactamase* dsRNA-injected cockroaches (negative control), the injection procedure proved not to affect on sex pheromone reception in the cockroach (19).

In control cockroaches (no-treatment and *β-lactamase* dsRNA-injected cockroaches), SSRs revealed that PA-SNs and PB-SNs in single *sw*-B sensilla exhibited reliable on-phasic responses to PA and PB, respectively (Fig. 2A). In contrast, in *PameORco* RNAi cockroaches, both PA responses of PA-SNs and PB responses of PB-SNs were significantly impaired (Figs. 2A–C and S3) (19). In *PameOR1* RNAi cockroaches, PB responses of PB-SNs remained unchanged compared with control cockroaches, but PA responses of PA-SNs were significantly impaired (Figs. 2A–C and S3). Conversely, in *PameOR2* RNAi cockroaches, PA responses of PA-SNs were unaffected, but PB responses of PB-SNs were significantly impaired. *PameOR53* and *PameOR62* RNAi had no detectable effects on PA and PB responses.

We confirmed the efficiencies of *PameOR1* and *PameOR2* RNAi *^Nymph^* by RT-qPCR (Figs. 1B and C; Table S2). In the RNAi cockroaches, the expression levels of *PameOR1* and *PameOR2* were significantly reduced to 33.2 ± 18.6% and 11.9 ± 5.9%, respectively. The RNAi efficiencies of *Pame*OR1 and *Pame*OR2 were lower than that of *Pame*ORco which were reduced to 1.5 ± 0.3% (Fig. 1D; Table S2) (19). Next, we characterized dose-dependent responses of PA-SNs to PA and PB-SNs to PB in each RNAi cockroach (Figs. 2D, E and S3). In control cockroaches, statistical analyses revealed that the response intensities of PB-SNs reached a plateau at 0.5 ng of PB, and the responses of PA-SNs reached a plateau at 0.2 ng of PA (Figs. 2D and E; Table S2). *PameOR1* and *PameORco* RNAi impaired the PA sensitivities of PA-SNs to the same degree. The PB sensitivity of PB-SNs was also significantly impaired in *PameORco* and *PameOR2* RNAi cockroaches, but PB-SNs of *PameOR2* RNAi cockroaches exhibited weak but reliable responses to high concentrations of PB.

To characterize the expression patterns of *PameOR1*, *PameOR2*, and *PameORco*, we performed *in situ* hybridization (ISH) experiments. ISH revealed a single *sw*-B sensillum has four OSNs expressing *PameORco* in line with the previous anti-*Pame*ORco labeling (Fig. 1E) (19), and one of four OSNs expressing *PameOR2* (Fig. 1F). Double fluorescence ISH also revealed that an OSN expressing *PameOR1* always paired with an OSN expressing *PameOR2* in the cell cluster of single *sw*-B sensillum, and there were no OSNs co-expressing both *PameOR1* and *PameOR2* (Fig. 1G). Taken together with anatomical and electrophysiological results, in sex pheromone-responsive *sw*-B sensilla, the *Pame*OR1/*Pame*ORco complex serves as the functional PA receptor in PA-SNs, and the *Pame*OR2/*Pame*ORco complex serves as the functional PB receptor in PB-SNs.

Our RNAi experiments confirmed tuning properties of PA-SNs and PB-SNs that have been implied in previous studies (19, 20); PA-SNs exhibited selective responsiveness to PA (Fig. S2D), whereas PB-SNs responded strongly to PB and weakly to PA (Fig. 1F). We clearly identified the PA sensitivities of PB-SNs by eliminating the PA responses of PA-SNs through *PameOR1* RNAi. The response sensitivities of PB-SNs to PA were lower than those to PB. PB-SNs reached a plateau at 2 ng of PA, and the response intensity to a sufficient concentration of PA was significantly weaker than that to PB (Figs. 2E and F). Furthermore, *PameOR1* RNAi also revealed that PB-SNs exhibited additive excitatory responses to PA mixed on PB (Fig. S4). These results suggest that PB is the best ligand for *Pame*OR2 in PB-SNs, but a sufficient concentration of PA can simultaneously activate both PA-SNs and PB-SNs.

RT-qPCR analysis and SSRs revealed long-lasting and prominent RNAi effects when dsRNA was injected into nymphal cockroaches (RNAi*^Nymph^*) (19). Therefore, RNAi*^Nymph^* cockroaches were used in subsequent experiments.

### The PB-processing pathway in the brain

**Fig. 3.**
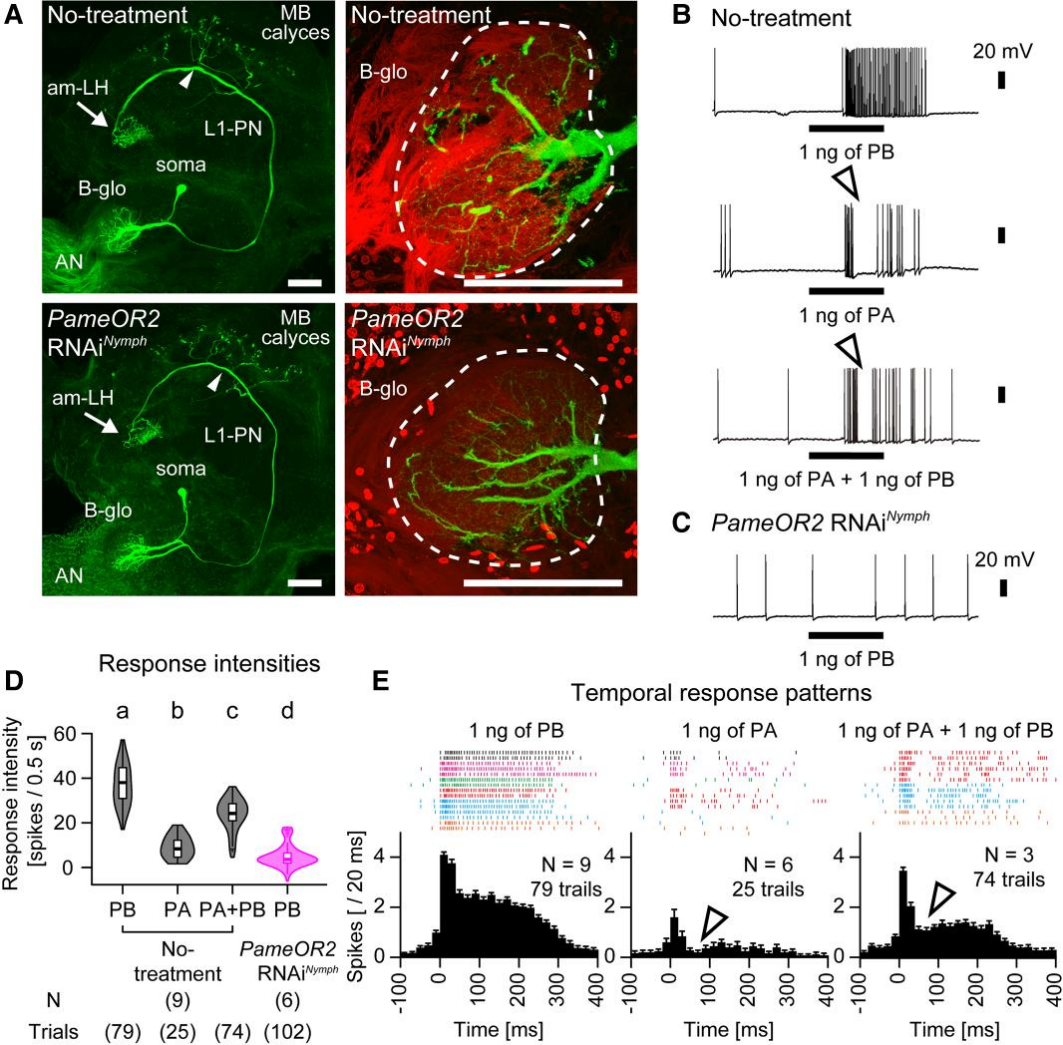
Sex pheromone responses of L1-PNs in no-treatment and *PameOR2* RNAi cockroaches. A) Morphology of L1-PNs in no-treatment (upper panels) and *PameOR2* RNAi*^Nymph^* (lower panels) cockroaches. L1-PN extends dendrites throughout the B-glomerulus (B-glo) and terminates ipsilaterally in both the mushroom body (MB) calyces and the antero-medial region of the lateral horn (am-LH). The arrowheads in the left panels indicate the insertion site of the glass electrode. AN, antennal nerve. Scale bar = 100 μm. B, C) Typical sex pheromone responses of L1-PNs in no-treatment B) and *PameOR2* RNAi*^Nymph^* C) cockroaches. D) Sex pheromone responses of L1-PNs in no-treatment and *PameOR2* RNAi*^Nymph^* cockroaches. Number of stimuli trials (Trials) and recorded L1-PNs (N) in no-treatment and *PameOR2* RNAi*^Nymph^* cockroaches are denoted in parentheses. Different letters above columns indicate significant differences (ANOVA with post hoc Tukey–Kramer test; *p* < 0.05). Median values with individual *P*-values are shown in Table S2. E) Temporal activity patterns of sex pheromone responses in L1-PNs from no-treatment cockroaches. The temporal activity pattern elicited by sex pheromones are displayed as raster plots and peri-stimulus time histograms with 20 ms bins. When L1-PN exhibits excitatory response, we regarded as the onset of the response to the spike whose instantaneous spike frequency of >150 Hz. In raster plots, sex pheromone responses from a given individual are denoted as the same color. In L1-PNs of no-treatment cockroaches, PA or PA + PB elicited the complex response with an on-phasic excitation followed by inhibition (white arrowheads in B and E). The histograms show means and +SEM, respectively. Number of trials of each olfactory stimulus and recorded L1-PNs are denoted in each of panels.

To investigate sex pheromone processing in brain neurons, we focused on the largest PN, L1-PN, which is one of multiple B-PNs (12–15 B-PNs) innervating the B-glomerulus where PB-SNs project to (26). L1-PN innervates throughout the B-glomerulus and projects axon terminals to specific regions of the ipsilateral MB calyces and LH via the medial antennal lobe tract (Fig. 3A). Since there is only one L1-PN in each brain hemisphere, it is readily identifiable across individuals. As is the case in *PameORco* RNAi (19), silencing *PameOR2* neither alters the afferent volume of the B-glomerulus or the overall morphology of L1-PN (Fig. 3A).

The L1-PNs in no-treatment cockroaches exhibited low but irregular spontaneous firing, and stimulation with 1 ng of PB evoked robust phasic–tonic responses outlasted the stimulus period (Figs. 3B–E), while those in *PameORco* RNAi cockroaches exhibited impaired activities in both spontaneous and PB stimulus periods (Figs. 3D and S5A) (19, 26). In *PameOR2* RNAi cockroaches, L1-PNs spontaneously fired at intervals of approximately 4 Hz, but PB stimulation rarely elicited action potentials (Figs. 3C, D and S5A). Consequently, *PameOR2* RNAi significantly impaired the PB responses of both PB-SNs and their downstream L1-PN.

Since PB-SNs responded not only to PB but also to PA, we examined the response of L1-PNs to PA in no-treatment cockroaches. Consistent with the tuning of PB-SNs, L1-PNs exhibited weaker on-phasic responses to PA compared with the same concentration of PB (Figs. 3B and E). Although both PA and PB activated PB-SNs, the postsynaptic L1-PN displayed significantly weaker responses to a mixture of PA and PB (PA + PB) stimuli compared with PB alone (Figs. 3B, D and E). The response pattern to PA + PB was complex in that inhibitory phases were inserted after the excitatory on-phasic phase (arrowheads in Figs. 3B and E). Moreover, PB-induced responses of L1-PNs were briefly interrupted after PA contacted to the antenna (Fig. S5). These results suggest the presence of interaction between the PA- and PB-processing pathways; the activation of L1-PN by PB might be suppressed by the activation of PA-SNs by PA through the interaction.

### Effects of the pheromone receptor knockdown on courtship behaviors in males

**Fig. 4.**
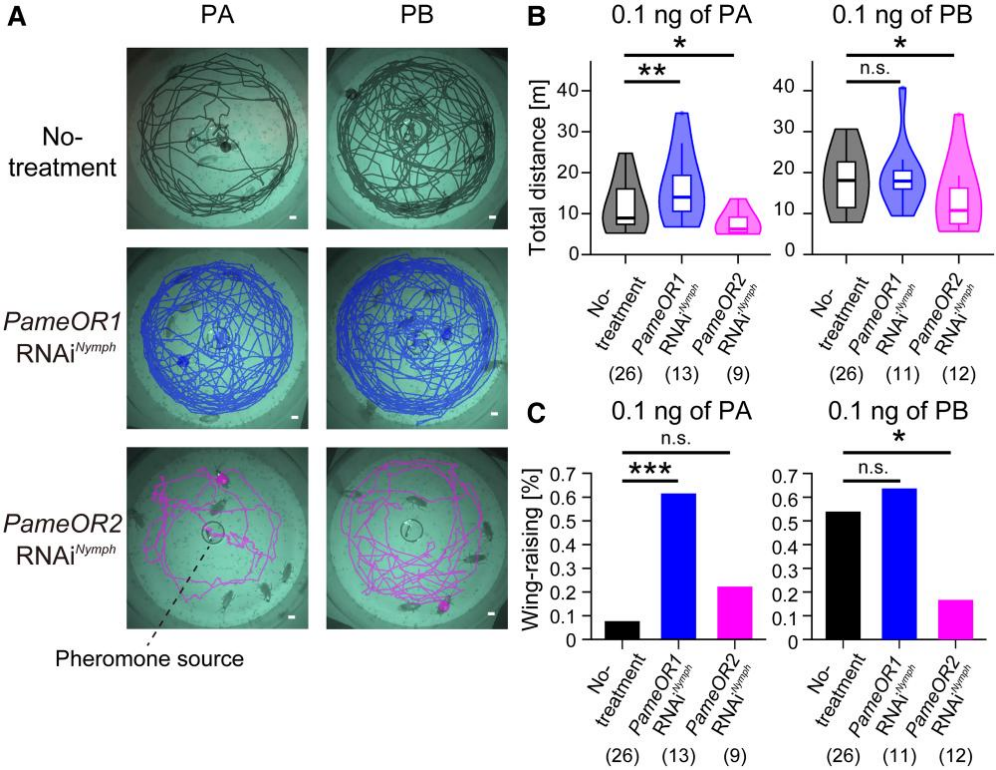
Effects of *PameOR1* or *PameOR2* RNAi on courtship behaviors elicited by PA or PB. A) Typical locomotion patterns of no-treatment and RNAi cockroaches in response to the PA or PB source. Tested cockroaches freely move within the round arena (30 cm diameter) during the experiment. The solid line in each panel shows the movement of a selected cockroach during the 4-min period after presentation of PA or PB (Videos S1 and S2). Scale bar = 1 cm. B) Total movement distance during the 4-min period after presentation of PA (left) or PB (right). Statistical differences are shown above each panel (Kruskal–Wallis test with post hoc Steel test; n.s., *p* > 0.05; *p < 0.05; **p <0.01). C) Percentages of cockroaches that exhibited wing-raising to PA (left) and to PB (right) in each group. Statistical differences are shown above each panel (Fisher's exact test; n.s., *p* > 0.05; *p < 0.05; **p <0.01). The number of tested cockroaches are shown in parentheses.

To investigate the functional aspects of interactive PA- and PB-processing pathways on cockroach courtship behaviors, we observed behavioral responses of *PameOR1* and *PameOR2* RNAi cockroaches to PA or PB. In behavioral experiments, no-treatment and RNAi male cockroaches were placed together in an arena (30 cm in diameter). We observed the behavioral responses of each male cockroach to PA or PB placed in the center of the arena (Figs. 4A and S6A). PA and PB were presented at the concentration of just above the behavioral threshold (0.1 ng; Figs. S6B and C). The movement of each cockroach was tracked during the 4-min period before and after presentation of PA or PB.

In the absence of sex pheromone, cockroaches from all three groups rarely moved within the arena (Figs. S6D and E). However, following exposure to PA or PB, the cockroaches immediately aroused to explore their surroundings, running along the wall of the arena and intermittently visiting the pheromone source with wing-raisings (Figs. 4, S6D–G and Videos S1 and S2). In no-treatment cockroaches, both PA and PB increased locomotion activities, but PB was more effective in eliciting long-distance movement and wing-raisings (Figs. 4B and C). When a PA + PB was presented after the 2-min period of PB presentation, no-treatment cockroaches tended to spend more time close to the PA + PB pheromone source (Fig. S7).

**Fig. 5.**
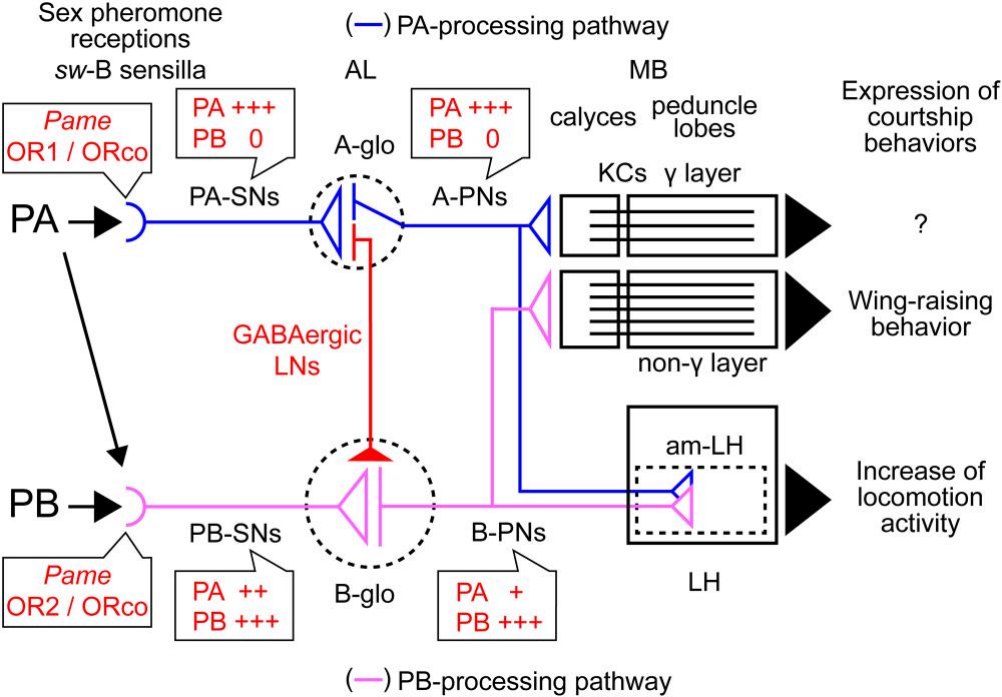
A schematic drawing of interactive parallel sex pheromone processing pathways controlling the courtship behaviors in the American cockroach. Our results suggest that the outputs of the PB-processing pathway play major roles in governing the expression of attraction and subsequent courtship behaviors, whereas the outputs of the PA-processing pathway only play minor roles. The PA- and PB-processing pathways, previously reported in other studies, are colored in blue and magenta, respectively (20, 22–24). Arrows from PA and PB denote tunings of PA- and PB-SNs to sex pheromones. The central interconnections between the PA- and PB-processing pathways are suggested in this study. Tunings functional sex pheromone receptors expressed in PA-SNs and PB-SNs, and the responsivities of sensory neurons and projection neurons comprising the PA- and PB-processing pathways are depicted in bubbles. Characters in bubbles are obtained from this study. The symbols “0”, “+”, “++”, and “+++” indicate levels of the response intensity and sensitivity, ranging from “no response” to “strong response with high sensitivity”.

*PameOR1* RNAi cockroaches, in which PA was selectively received by PB-SNs, had higher locomotion activities in response to PA than no-treatment cockroaches (Figs. 4B and S6F). In contrast, *PameOR2* RNAi cockroaches, in which PA was selectively received by PA-SNs, exhibited significantly weaker locomotion activities to PA than no-treatment cockroaches (Figs. 4B and S6F). In the presence of PA, *PameOR1* RNAi cockroaches frequently exhibited wing-raisings during moving around the arena compared with no-treatment cockroaches (Fig. 4C). *PameOR2* RNAi cockroaches displayed significantly reduced locomotion activities and wing-raisings in response to PB compared with no-treatment cockroaches (Figs. 4B, C and S6G). Knockdown of *PameOR1* did not affect the locomotory activities and wing-raisings elicited by PB. Taken together, results from behavioral and electrophysiological experiments suggest that the activation of the PB-processing pathway is crucial for the expression of courtship behaviors, and PA suppresses the expression of courtship behaviors by inhibiting the activation of the PB-processing pathway (Fig. 5).

## Discussion

In the cockroach, courtship behaviors can be induced by either the major sex pheromone component PB or the minor component PA, but PA elicits a weaker behavioral response than PB (14, 15). However, when PA is presented together with PB, PA suppresses behaviors induced by PB in a dose-dependent manner (14, 17). Thus, the minor component PA has promotive and suppressive functions in courtship behaviors depending on the pheromonal context. In this study, we identified the PA and PB receptors and their ligand specificities in the cockroach for the first time. By combining knockdown of the sex pheromone receptor genes with electrophysiological or behavioral experiments, we characterized neural circuits important for courtship behaviors in the cockroach (Fig. 5). We revealed that PB-SNs and postsynaptic B-PNs were activated not only by PB but also by PA, which resulted in an increase in locomotion activities and courtship behaviors (PB-processing pathway: magenta lines in Fig. 5). By contrast, PA-SNs and A-PNs were selectively activated by PA (23), and it elicited male cockroach locomotor activity without triggering the courtship behaviors (PA-processing pathway: blue lines in Fig. 5). At the same time, the activation of the PA-processing pathway inhibited the activation of B-PNs. The physiological interactions between the PA- and PB-processing pathways highlight the promotive and suppressive functions of the minor pheromone component PA (Fig 5).

We identified PA and PB receptors in *P. americana* as heteromeric complexes: *Pame*OR1/*Pame*ORco and *Pame*OR2/*Pame*ORco, respectively (Fig. 2). Phylogenetic analysis of *PameORs* has revealed that *PameOR1* and *PameOR2* belong to the same clade, which is distinct from the other *PameORs* clades, and the clade exhibits lineage-specific expansions in Blattodea insects, including *P. americana*, *Zootermopsis nevadensis*, and *Blattella germanica* (28, 29). These results suggest that the PA and PB receptors have evolved specifically in Blattodea insects.

*Pame*OR1 was specifically tuned to the minor component PA, while *Pame*OR2 exhibited broader tuning to both the major PB and minor PA, albeit with lower sensitivity to PA compared with PB. This differs from general model insects for sex pheromone processing, such as *Drosophila melanogaster* (30), *Manduca sexta* (31, 32), and *Bombyx mori* (10), where the major sex pheromone component is exclusively received by the specific receptor expressed in the specific sensory neurons and processed by an independent pathway. Consequently, in these insects, the minor sex pheromone component alone does not elicit courtship behaviors. However, in the cockroach, a sufficient concentration of the minor component PA can activate both PA-SNs and PB-SNs and elicit courtship behaviors (14, 15, 17). Because our results indicate that activation of the PB-processing pathway is critical for the expression of courtship behaviors, the loose ligand specificity in *Pame*OR2 contributes to the promotive function of PA (Fig. 5).

Among 12–15 B-PNs innervating the B-glomerulus, L1-PN serves as an electrophysiological model neuron for studying sex pheromone processing in the cockroach (23, 26, 33, 34). L1-PN exhibits excitatory responses to both PA and PB, indicating that it is closely correlated with presynaptic PB-SNs. PB elicited phasic–tonic excitatory responses that outlasted the stimulus period in L1-PN, while PA or PA + PB elicited the complex response with an initial excitation followed by inhibition (Figs. 3B and E), which is similar to responses in PNs arborizing in ordinary glomeruli (35). The complex responses in ordinary PNs are mediated by GABAergic local interneurons (LNs) that connect multiple glomeruli (35, 36), and electronmicroscopic observations have revealed numerous input synapses from GABAergic LNs onto the dendrites of L1-PN (37). A sufficient concentration of PA strongly activates PA-SNs and weakly activates PB-SNs at same time. Therefore, in response to PA or PA + PB, L1-PN might first receive direct excitatory inputs from PB-SNs and then receive strong inhibitory inputs from GABAergic LNs via the A-glomerulus where PA-SNs project (Fig. 5). Indeed, we observed significant inhibition to contact PA during PB presentation (Fig. S5). A-PNs selectively respond to PA but not PB (23, 24, 38), which is consistent with the responsivity of PA-SNs. These findings, combined with the results of previous studies, clarify the inhibitory interaction from the PA-processing pathway to the PB-processing pathway in the AL (Fig. 5).

It has been known that OSNs colocalized within a given sensillum exhibit nonsynaptic reciprocal inhibition through ephaptic coupling when they are simultaneously activated (39, 40). Because PA activates both PA- and PB-SNs co-housed in single *sw*-B sensilla, PA or PA + PB may induce ephaptic inhibition between PA-SNs and PB-SNs. Inhibition between sex pheromone-responsive neurons co-housed in the same sensillum was previously reported in several heliothine moth species (41, 42). In general, the morphologically larger OSN with larger amplitude spikes in a pair corresponds to the dominant neuron in ephaptic inhibition (40). The similar amplitudes of PA- and PB-SNs make it difficult to analyze ephaptic inhibition in the cockroach pheromone reception. On-phasic excitation and the subsequent inhibition of L1-PNs to PA or PA + PB strongly imply the inhibitory interconnection from the A-glomerulus to the B-glomerulus in the AL, but we cannot exclude the possibility that the peripheral ephaptic inhibition between PA- and PB-SNs influences on the weaker response of L1-PNs to PA or PA + PB.

The interactive PA- and PB-processing pathways explain the courtship behaviors elicited by PA or PB in pheromone receptor knockdown cockroaches (Fig. 5). In the no-treatment cockroach, PB is selectively received by *Pame*OR2 and activates PB-SNs and postsynaptic B-PNs, leading to typical courtship behaviors. Conversely, *PameOR2* RNAi cockroaches, in which activations of PB-SNs and B-PNs were impaired, showed significantly reduced locomotion activities and courtship behaviors triggered by PB. In *PameOR1* RNAi cockroaches, PA activated PB-SNs via *Pame*OR2, which resulted in the expression of typical courtship behaviors. These findings strongly indicate that the activation of the PB-processing pathway is crucial for the expression of courtship behaviors in the cockroach. In no-treatment cockroaches, PA activated both PA-SNs and PB-SNs, but activations of B-PNs is suppressed by activations of PA-SNs. Therefore, *PameOR1* RNAi cockroaches, in which PA selectively activated the PB-processing pathway without inhibitory effects, expressed stronger courtship behaviors in response to PA compared with no-treatment cockroaches.

The sex pheromone processing pathways proposed here raise questions regarding the function of the activation of the PA-processing pathway. In *PameOR2* RNAi cockroaches, PA selectively activates the PA-processing pathway, which arouse the cockroach activity but does not trigger courtship behaviors (Figs. 4 and S6). Increased locomotor activity was also observed with the selective activation of the PB-processing pathway (PA or PB responses in *PameOR1* RNAi cockroaches in Figs. 4 and S6). The axon terminals of A-PNs and B-PNs are convergent in the antero-medial region of the LH (Fig. 3A) (24, 25), suggesting that this specific region might control the initiation of common pheromone searching behaviors (Fig. 5). Courtship behaviors, such as wing-raisings, were exclusively elicited by the activation of the PB-processing pathway and might be regulated by segregated pathways in the higher center. A-PNs and B-PNs are known to terminate in different regions in the MB calyces and likely synapse on intrinsic KCs of different morphological types (24, 26).

Under natural conditions, an adult female cockroach releases a mixture of PA and PB at a ratio of approximately PA:PB = 1:10 from the colon (43–45). Behavioral experiments using a wind tunnel reveal that male cockroaches are attracted to PA or PB presented alone, but PB is approximately 30 times more effective than PA (16, 46). Therefore, long-distance attraction of males is possible with PB, but not with PA (46). However, female feces and the resident shelters contain a higher concentration of PA at a ratio of approximately PA:PB = 8:5 (45, 47), and PA has been suggested to play role in locating mates at relatively short distances from females (17). Therefore, when an adult male is positioned far away from the pheromone source, the sex pheromone predominantly activates the PB-processing pathway by PB, which cause the male to orient towards the pheromone source with wing-raisings. In fact, male adult cockroaches can accurately locate the PB source without PA (17, 48). As the male approaches the female, the minor component PA activates PA-SNs in a dose-dependent manner and suppresses the activation of the PB-processing pathway. This likely results in changes in cockroach behavior; cockroaches decrease their locomotion activities and remain in close contact with the pheromone source. It is corresponding with the result of our behavioral experiments that no-treatment cockroaches aroused by PB tend to stay the pheromone source where a mixture of PA and PB presented (Fig. S7). Although future work needs to investigate the courtship behaviors elicited by various pheromonal composition ratios, this hypothesis can explain the attractive function of PB and the arrestive function of PA for the cockroach courtship behaviors.

To localize the pheromone source, a recent behavioral study has suggested that walking cockroaches primarily use the spatial distribution of sex pheromones, whereas flying moths predominantly use the timing of pheromone encounters (49). This has led to the suggestion that strategies for sex pheromone processing differ between these insects. In the moth, each pheromone component is independently processed by specific pathways from the periphery to higher brain centers (8, 9, 50). For moths to locate conspecific females, all pheromone components need to be emitted in the right ratio (3, 6, 51), and the central brain region must decode the combinational activities of PNs from the simultaneously activated glomeruli representing the key pheromone component blend (7, 52–54). In moths, GABAergic local interneurons in the AL control the synchronization of spike firing across PNs from different macroglomeruli (52, 55), and the responses of PNs processing the major sex pheromone component are rarely affected by the co-presented minor sex pheromone component (11, 56). Therefore, moths have been hypothesized to have neural circuits within the lateral protocerebrum that govern the suppression of courtship behaviors by the minor component (11, 57, 58). By contrast, in the cockroach, the ratio of pheromone components is less of a priority; PB and PA predominantly work at long and short distances from the pheromone source, respectively (17, 48). By detecting changes in pheromone context in the environment through interactions between the PB- and PA-processing pathways, the cockroach dynamically alters its behavior and correctly locates the pheromone source.

## Materials and methods

Procedures for molecular cloning, RT-qPCR, RNA interference, *in situ* hybridization, fluorescence *in situ* hybridization, intracellular recording, and staining of L1-PN are described in Supplementary Methods.

### Insects

Last instar (LI: 11th instar) and adult males of the cockroaches *P. americana*, with intact antennae, were obtained from laboratory colonies maintained at 27 ± 1°C under a 12:12 light–dark cycle at Fukuoka University. LI nymphs were identified based on the lengths of the body and hind tibia (59). Cockroaches were collected from colonies immediately after molting and individually reared.

### SSRs from sex pheromone-responsive *sw*-B sensilla

An ice-anesthetized cockroach was immobilized ventral-side-up on an acrylic plate, and then the body, legs, and antennae were gently fixed using low melting wax. We selected *sw-*B sensilla on the ventral surface of the antenna under a light microscope at a magnification of 500× (AZ100, Nikon, Tokyo, Japan). A tungsten indifferent electrode was manually inserted into the head capsule near the ipsilateral compound eye. Using a micromanipulator, the tip of the glass electrode filled with cockroach saline was carefully inserted into the basal cavity of a *sw-*B sensillum. After observation of the spontaneous activities of OSNs, sex pheromones were presented to the antenna. The electrical signals were amplified and processed through a preamplifier (MEZ-8201; Nihon Kohden, Tokyo, Japan) and a main AC/DC amplifier (EX-1; Dagan Corporation, Minneapolis, MN, USA), then displayed on an oscilloscope. The AC signals were filtered (bandpass 100 Hz to 1 kHz) and recorded with a Power Lab data acquisition system at a sampling rate of 10 kHz (Power Lab 8/35; AD Instruments Japan Inc., Nagoya, Japan). In each animal, we repeatedly recorded olfactory responses from different *sw-*B sensilla. Spikes of PA-SNs and PB-SNs were sorted and counted using the spike sorting function of Spike 2 ver. 8.08 (CED, Cambridge, UK).

### Sex pheromone stimulation

We used synthetic pure *P. americana* sex pheromones, PA and PB (60, 61). PA and PB were prepared by diluting them in hexane at arbitrary concentration. Each of the sex pheromone solutions was applied onto an aluminum plate (15 × 5 mm) and left to dry for >1 min. Therefore, their concentrations are denoted here as dry weights. Just before the recordings, two aluminum plates containing PA or PB were separately inserted into two glass pipettes.

Because of the difficulty of discriminating small-amplitude spikes from PA-SN and PB-SN according to their spike shapes (Figs. S2A and B), PA responses of PA-SNs and PB responses of PB-SNs were characterized by performing SSRs with a cross-adaptation odor stimuli system (Figs. S2C and D) (19). Fresh air was cleaned and dried by passing through charcoal and silica-gel filters. The airflow rate in the main tube was maintained at 1 L/min using a flowmeter. The main tube was tandemly connected to two electric-driven three-way solenoid valves (Fig. S2C), which were independently operated. During the inter-stimulus period, the constant airflow passing through both valves and the empty glass pipette was directed over the antenna. A glass pipette containing PB and that containing PA were connected to the outlets of Valve 1 and Valve 2, respectively. The tips of two glass pipettes were positioned approximately 1 cm apart from the recording sensillum. The recording sensillum received three consecutive PB stimuli, followed by a PA stimulus, with inter-stimulus intervals of 0.5 s (Fig. S2D).

The response intensities of PA-SN and PB-SN to PA and PB were determined by calculating the increase in the total spike frequency compared with the spontaneous frequency as follows: R—R0, where R0 and R represent the total number of spikes during the 0.5 s period before and after the onset of stimulation, respectively. Statistical analysis was performed using one-way or two-way ANOVA with post hoc Tukey–Kramer test using BellCurve for Excel (Social Survey Research Information, Tokyo, Japan) and R software (4.3.0; https://www.r-project.org/).

### Behavioral experiments and analysis

Behaviors of the cockroaches to PA or PB were assessed in a circular arena with a diameter of 30 cm (Fig. S6A). To prevent animals from climbing, the arena wall (10 cm in height) was coated with liquid paraffin. A filter paper was placed underneath the arena. Multiple paper shelters were randomly placed within the arena, and a petri dish (3.5 cm in diameter) containing food pellets was positioned at the center. Six or four virgin 7-day-old males, consisting of the equivalent numbers of no-treatment and RNAi*^Nymph^* cockroaches, were introduced into the arena. To accommodate the experimental conditions, the cockroaches in the arena fed foods and water at ad libitum for 4–7 days. The temperature was kept at 27°C.

At 3 h before the behavioral experiments, the paper shelters, food pellets, and water were removed from the arena. The initial phase of the behavioral experiment involved observing the cockroaches for 4 min without any stimuli (Fig. S6). Subsequently, an aluminum plate (15 × 5 mm) loaded either 0.1 ng of PA or PB was gently placed into the petri dish. Because the arena is small for *P. americana*, our experimental setup was designed to mimic the situation where males are placed close to a female. The behavioral responses of each tested cockroach to the presented sex pheromone were recorded during a 4-min post-stimulus period. The behavioral experiment was performed in the scotophase, and the behavioral responses were captured using an infrared camera with at 30 fps (FDR-AX60, Sony, Tokyo, Japan). After recording, the paper shelters, food pellets, and water were reintroduced into the arena. Another session of the behavioral experiment with a different sex pheromone was performed after a minimum of 4 days from the first session. Following the second session of the experiment, the antennae of each RNAi cockroach were removed and homogenized in TRIzol Reagent for RT-qPCR analysis.

The behavior of each tested cockroach was analyzed using UMAtracker, an open-source software designed for tracking animals (http://ymnk13.github.io/UMATracker/#). The position of each cockroach within the arena was automatically tracked and converted to X and Y coordinates every five frames. To assess the locomotion activities, we quantified the total movement distance of each cockroach during the 4-min period before and after the presentation of the sex pheromone. Additionally, we manually counted the number of cockroaches exhibiting a wing-raising response to the sex pheromone during the same 4-min period. During the sequence of the courtship behavior, male cockroaches exhibit two different wing-raisings, partial and full wing-raisings (5). Because it is difficult to divide them in our small experimental arena, the wing-raising behavior mentioned in this article was defined without distinguishing between partial and full wing-raisings. The wing-raising ratio (%) of the RNAi cockroaches was calculated as follows: the number of cockroaches expressing wing-raising/ the number of tested cockroaches. We also calculated the time of each cockroach staying in close to the pheromone source (7 cm in diameter of the center of the petri dish) as a staying time. Statistically comparisons of locomotion activities and wing-raising ratios were performed among the no-treatment, *PameOR1* RNAi, and *PameOR2* RNAi cockroaches using the Kruskal–Wallis test with post hoc Steel test for locomotion activities (n.s., *p* > 0.05; *p < 0.05; **p <0.01) and Fisher's exact tests for wing-raising ratios (*p < 0.05; ***p <0.001). In each cockroach, the staying time during the first 2-min period after the sex pheromone presentation was compared that during the following 2-min period using the Wilcoxon signed-rank test. Detailed results of statistical analysis were summarized in Table S2.

## Supplementary Material

pgae162_Supplementary_Data

## Data Availability

All data are included in the manuscript and Supplementary materials. The nucleotide sequences of the obtained cDNA are available at GenBank (*PameOR1*; LC781791, *PameOR2*; LC781792, *PameOR53*; LC781793, and *PameOR62*; LC781794). This article does not report original codes.
