# Supplementary material for: Interactive parallel sex pheromone circuits that promote and suppress courtship behaviors in the cockroach
Source: PNAS Nexus. 2024 Apr 15;3(4):pgae162. doi: 10.1093/pnasnexus/pgae162 (PMC11058470; doi:10.1093/pnasnexus/pgae162)
Supplement: pgae162_Supplementary_Data [file pgae162_supplementary_data.zip › PNASNEXUS-PNASNEXUS-2023-01140-TR-s01.docx]

**
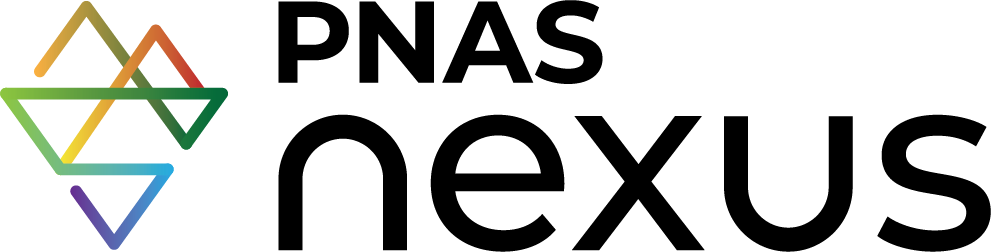
**

**Supplementary Information for**

**Interactive parallel sex pheromone circuits that promote and suppress courtship behaviors in the cockroach**

Kosuke Tateishi^1,2^, Takayuki Watanabe^3^, Mana Domae^4^, Atsushi Ugajin^5^, Hiroshi Nishino^4^, Hiroyuki Nakagawa^1^, Makoto Mizunami^4^, and Hidehiro Watanabe^1^*

1. Department of Earth System Science, Faculty of Science, Fukuoka University, Fukuoka 814-0180, Fukuoka, Japan

2. School of Biological and Environmental Sciences, Kwansei Gakuin University, Sanda 669-1330, Hyogo, Japan

3. Research Center for Integrative Evolutionary Science, The Graduate University for Advanced Studies Shonan Village, Hayama 240-0193, Kanagawa, Japan

4. Research Institute for Electronic Science, Hokkaido University, Sapporo 060-0812, Hokkaido, Japan

5. JT Biohistory Research Hall, Takatsuki 569-1125, Osaka, Japan

*Correspondence:

Dr. Hidehiro Watanabe

E-Mail: [nabehide@fukuoka-u.ac.jp](mailto:nabehide@fukuoka-u.ac.jp)

**This PDF file includes:**

Figures S1 to S7

Tables S1 and S2

Legends for Videos S1 and S2

**
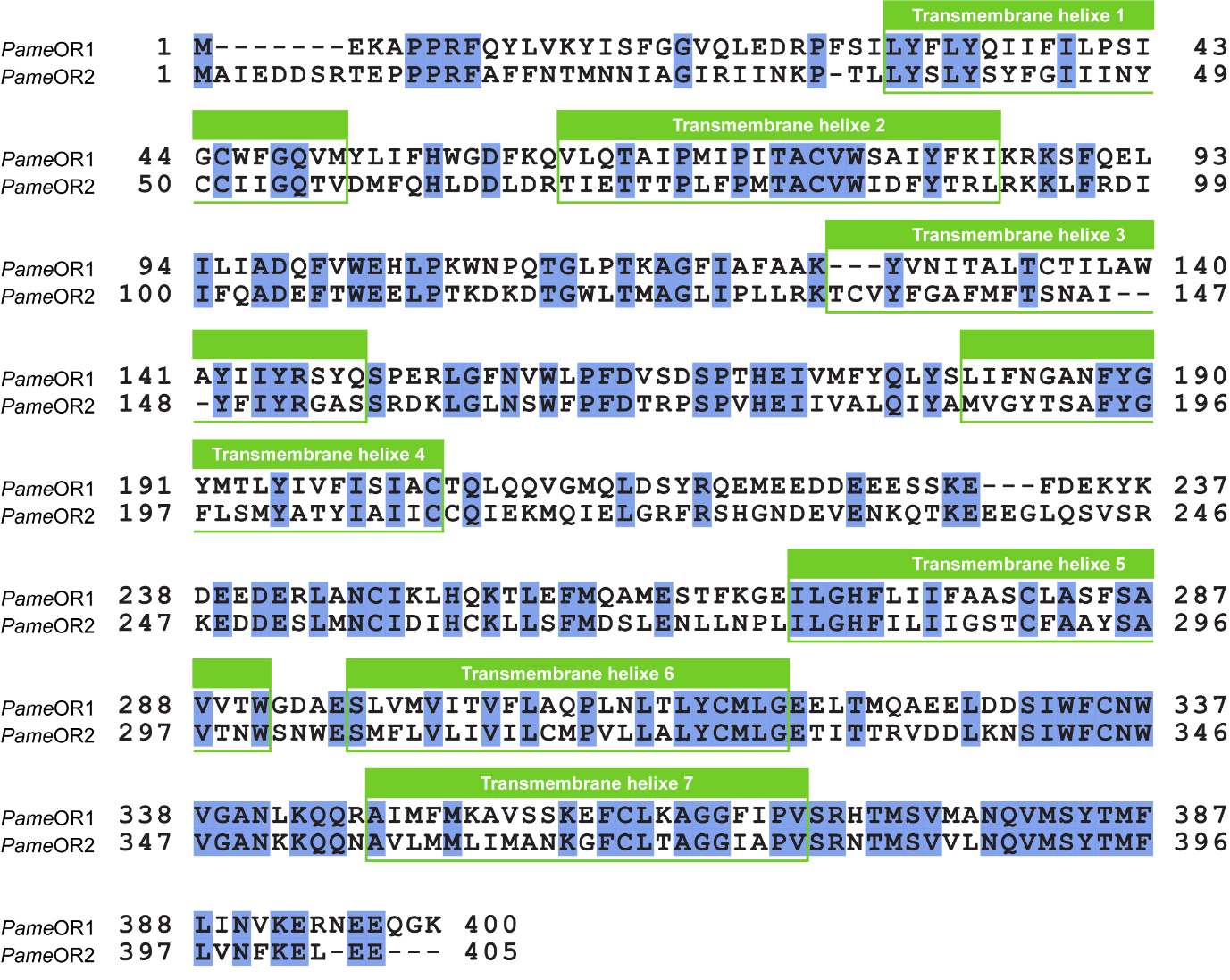
**

**Fig. S1. Alignment of amino acid sequences of two sex pheromone receptors (*Pame*OR1 and *Pame*OR2) in *Periplaneta americana*.** Deduced amino acid sequences of *Pame*OR1 and *Pame*OR2 were aligned. The letters are shaded by alignment strength. The positions of seven transmembrane helixes are predicted as illustrated. GenBank IDs: *PameOR1*; LC781791; and *PameOR2*; LC781792.

**
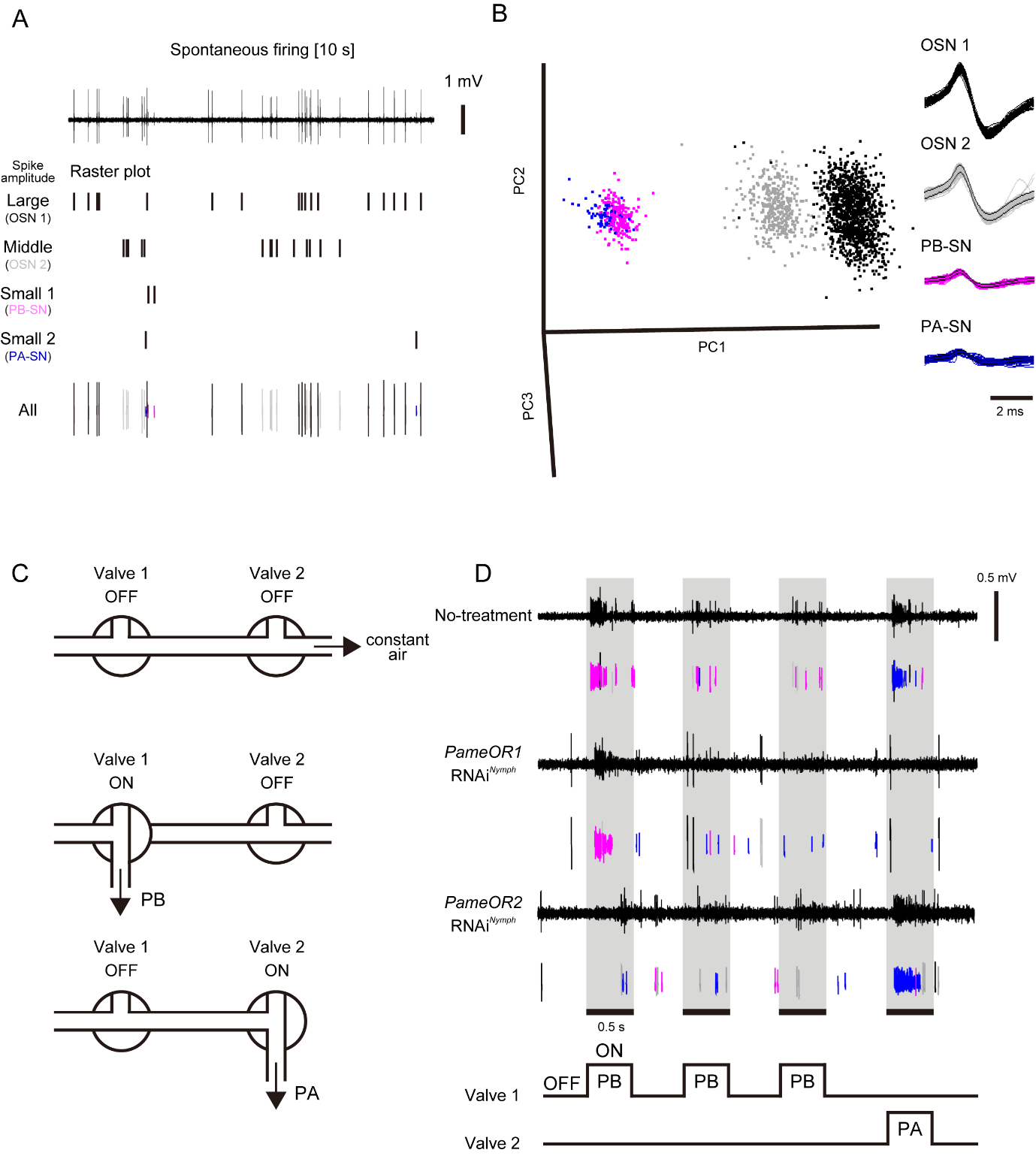
**

**Fig. S2. Identification of PA responses of PA-SNs and PB responses of PB-SNs in single *sw*-B sensilla** (A, B) Segregation of spikes of four OSNs in a *sw*-B sensillum. Typically, the electrophysiological trace from a single *sw*-B sensillum exhibits large, middle and two small amplitude spikes (A). Large spikes of OSN 1 (black) and middle spikes of OSN 2 (gray) are clustered in the 3D-plot based on the first three principal components (PC1-PC3) obtained from the principal component analysis of spike shapes, whereas small spikes from PA-SN (blue) and PB-SN (magenta) cannot be segregated (B). (C, D) A cross-adaptation odor stimuli system. To segregate PB responses of PB-SN and PA responses of PA-SN, we developed the cross-adaptation odor stimuli system. The glass pipette containing PB and that containing PA were connected to the outlets of Valve 1 and Valve 2, respectively (C). During the sex pheromone stimuli period, the recording sensillum received three successive PB stimuli and followed one PA stimulus with inter-stimulus intervals of 0.5 s. Each sex pheromone stimulus was presented for 0.5 s (D). PB responses of a PB-SN were adapted by repeated stimuli (no-treatment in D). This system enables us to precisely evaluate PA responses of the PA-SN and PB responses of the PB-SN in sex pheromone receptor RNAi cockroaches. Images shown in A, B, and C are obtained from Tateishi et al., 2022.

**
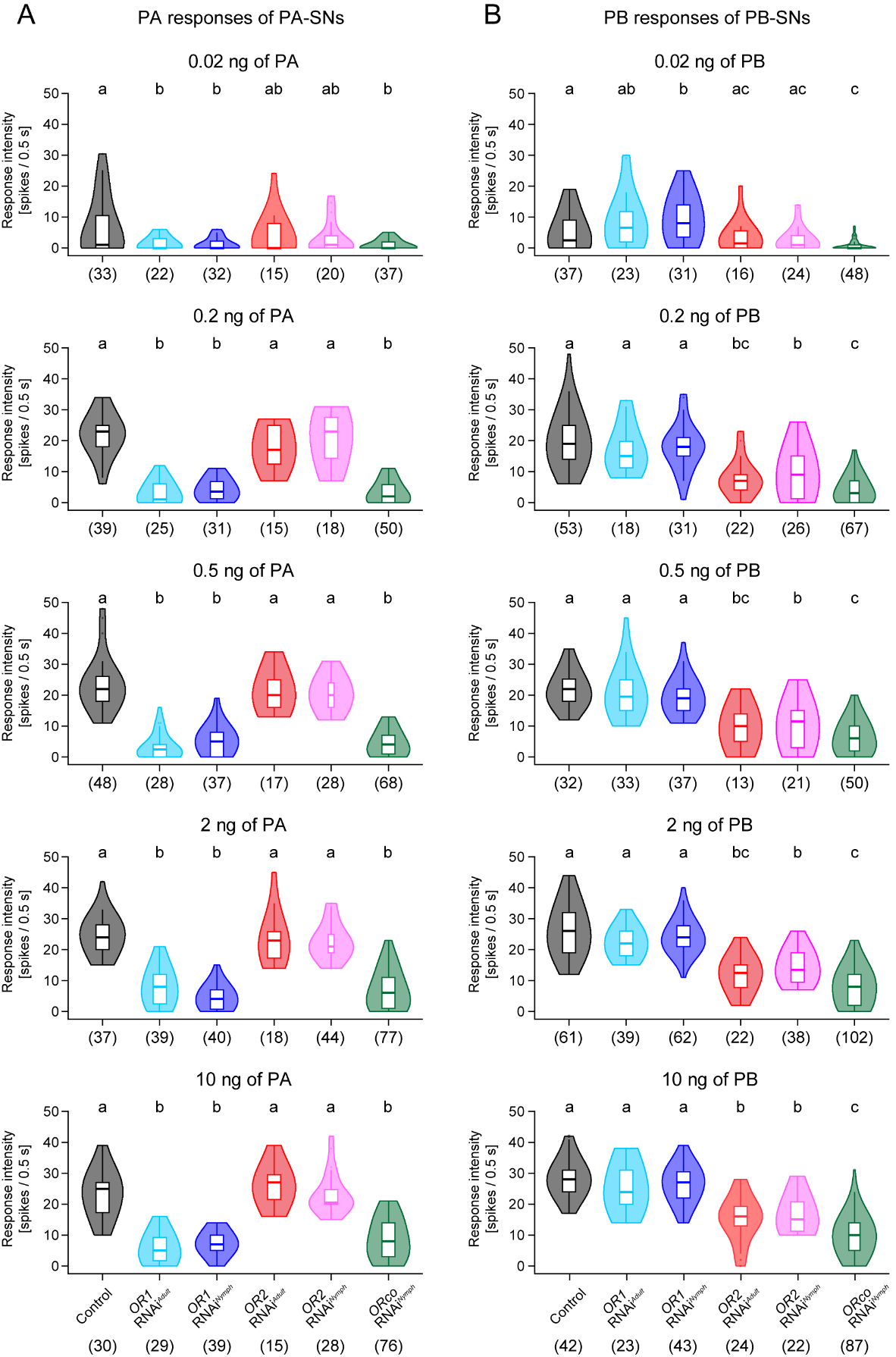
**

**Fig. S3. Dose-responses of PA-SNs and PB-SNs in naïve and RNAi cockroaches.** (A) Responses of PA-SNs to a given concentration of PA. (B) Responses of PB-SNs to a given concentration of PB. Responses were obtained from no-treatment and five different RNAi cockroaches (*PameOR1^Nymph^*, *PameOR1^Adult^*, *PameOR2^Nymph^*, *PameOR2^Adult^* and *PameORco^Nymph^*). The different letters above each plot indicate significant differences (ANOVA post-hoc Tukey-Kramer test; p < 0.05).

**
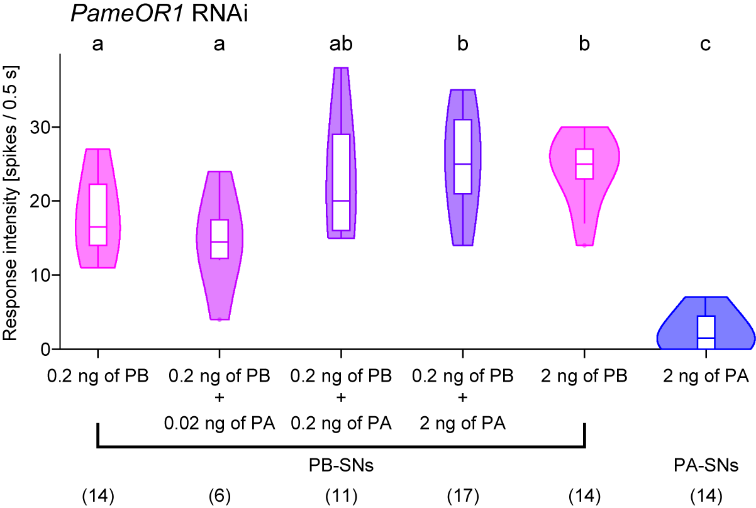
**

**Fig. S4. Responses of PB-SNs to sex pheromone mixtures.** By silencing the PA responses of PA-SNs using *PameOR1^Nymph^* cockroaches, response of PB-SNs to the mixture of PA and PB were obtained. The different letters above each plot indicate significant differences (ANOVA post-hoc Tukey-Kramer test; p < 0.05).

**
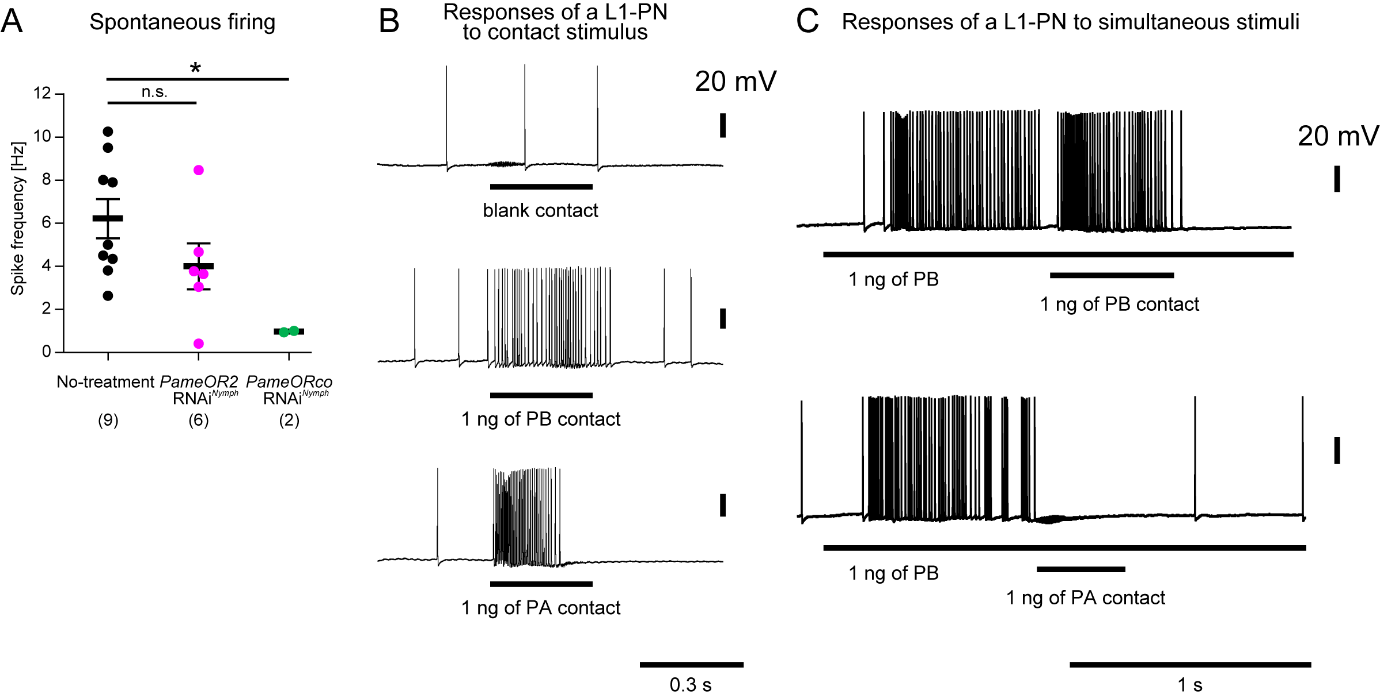
**

**Fig. S5. Responses of L1-PNs to contacts of sex pheromones.** (A) Spontaneous activities of L1-PNs in no-treatment, *PameOR2* RNAi*^Nymph^*, and *PameORco* RNAi*^Nymph^* cockroaches. The sample number is noted in parentheses and black bars indicate means ± SEM. Statistical differences are shown above the graph (Kruskal-Wallis post-hoc Steel test; n.s = p > 0.05, * = P < 0.05) Spontaneous activities of L1-PNs from *PameORco* RNAi*^Nymph^* cockroaches were obtained from Tateishi et al. (2022). (B) Responses of a L1-PN to contact presentation of PA or PB in no-treatment cockroaches. A pheromone-immersed filter paper (tip width: 1 mm) was manually put in contact with the middle region of the antennal flagellum. The stimulus timing was monitored by a dynamic-strain measuring device. (C) Responses of a L1-PN to contact presentation of PA or PB during the excitatory phase elicited by olfactory PB stimuli. The excitatory responses of a L1-PN to olfactory PB stimuli were suppressed by the contact PA stimuli but not by the contact PB stimuli.

**
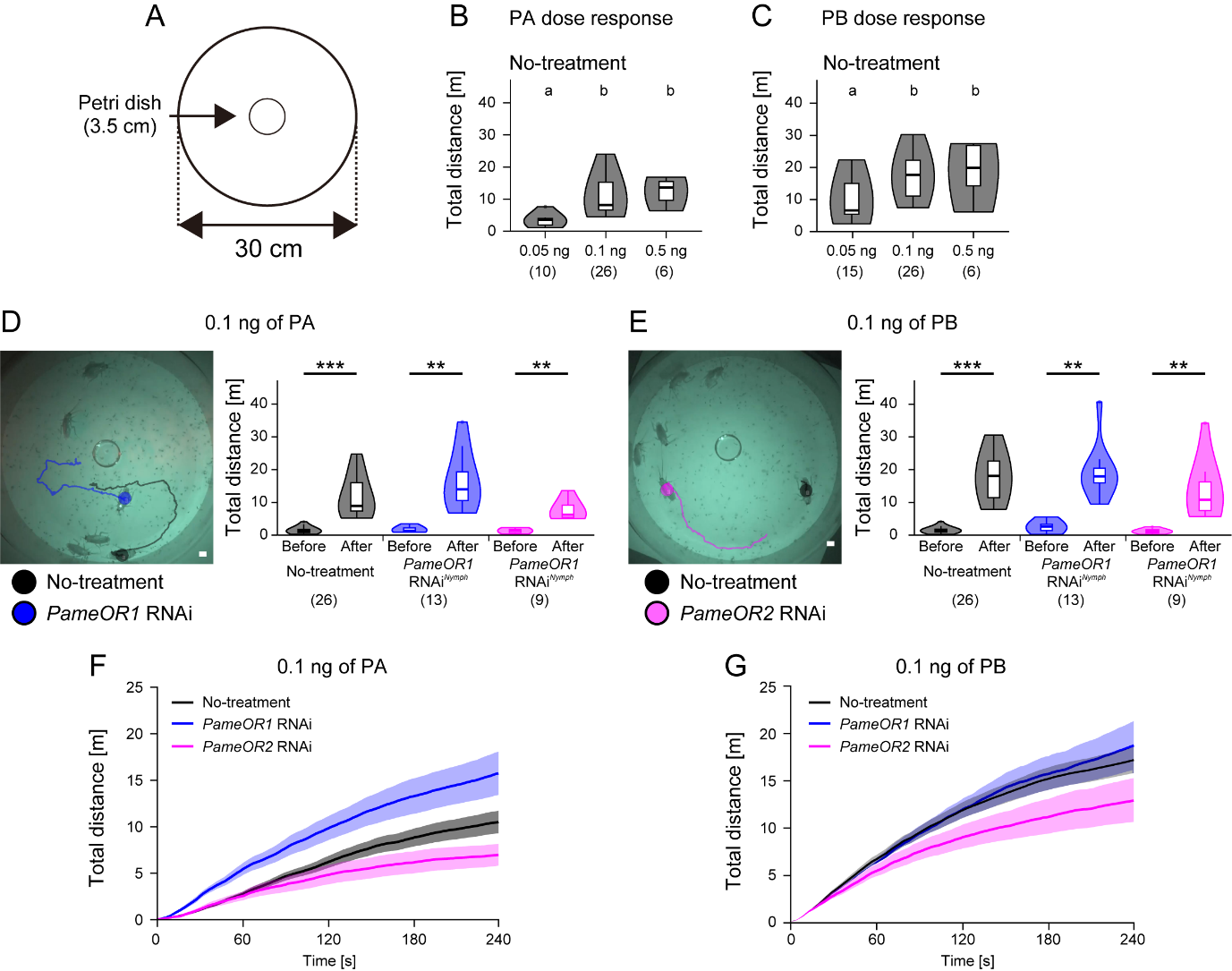
**

**Fig. S6. Locomotion activities of no-treatment and *PameORs* RNAi cockroaches before and after sex pheromones presentation.** (A) Schematic drawing of the experimental arena. Tested cockroaches freely moved within the round arena (30 cm diameter) during the experiment. (B, C) Locomotion activities of no-treatment cockroaches elicited by different concentration of PA (B) and PB (C). The different letters above each plot indicate significant differences (ANOVA post-hoc Tukey-Kramer test; p < 0.05). (D, E) Typical locomotion patterns of no-treatment and RNAi cockroaches before presentation of sex pheromones. The solid line in each panel shows the movement of a selected cockroach during the 4-min period before presentation of sex pheromones. Black line: No-treatment cockroach, blue line: *PameOR1* RNAi cockroach, magenta line: *PameOR2* RNAi cockroach. Total movement distance during the 4-min period before and after presentation of 0.1 ng of PA (D) or PB (E). In all groups, PA and PB activated cockroach locomotions. Statistical differences are shown above each panel (Wilcoxon signed-rank sum test: *** = P < 0.001, ** = P < 0.01). (F, G) Cumulative movement distances of no-treatment and RNAi cockroaches after presentation of PA (F) and PB (G). The solid lines and shaded areas indicate means and ± standard errors, respectively.

**
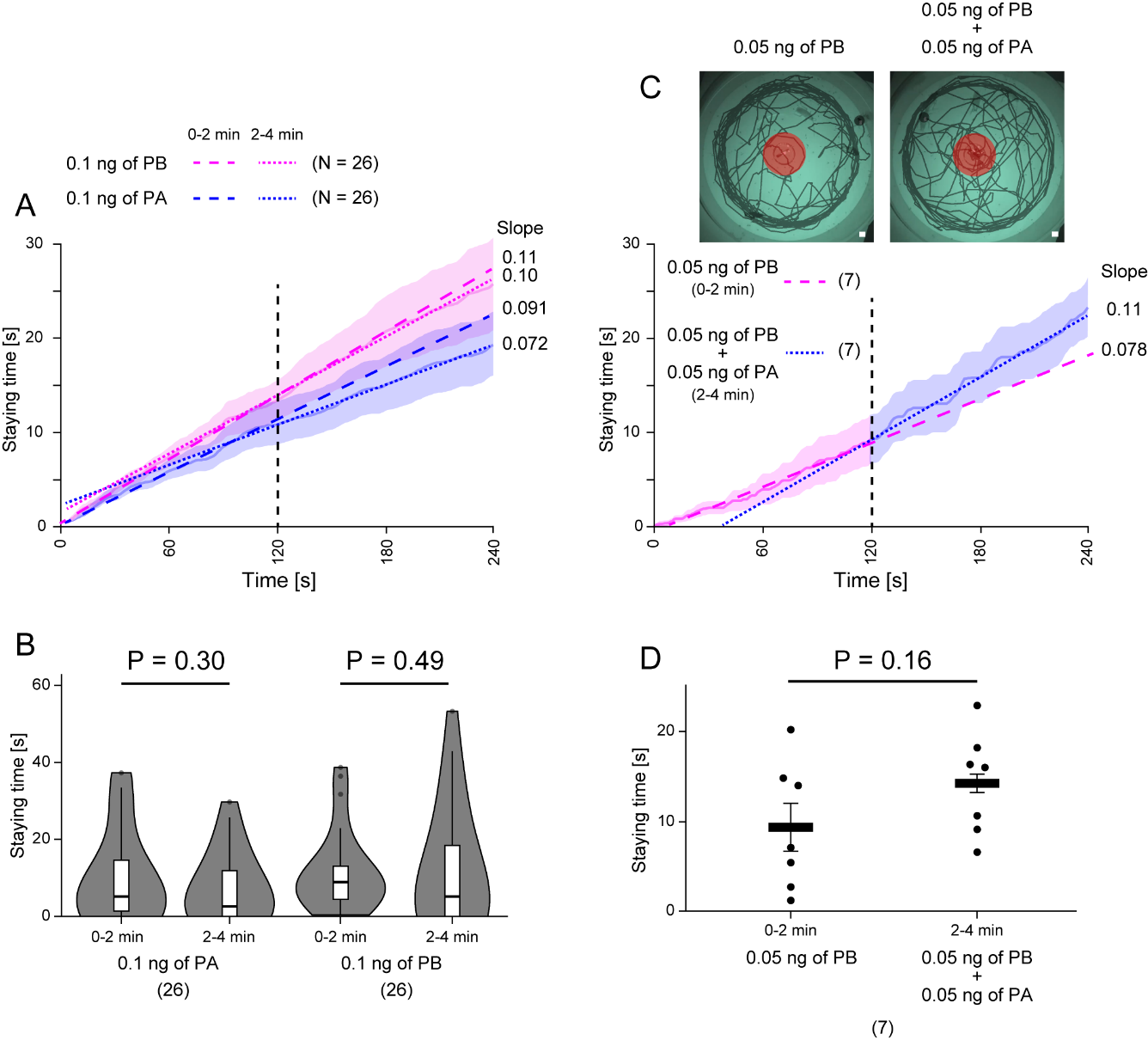
**

**Fig. S7. Staying times at the pheromone source in no-treatment cockroaches.** (A, B) Staying times at the PA or PB source of no-treatment cockroaches. For each cockroach, the time spent in close to the pheromone source (within a 7 cm in diameter from the center of the petri dish) was calculated as the staying time. Changes of staying times during 4-min period of 0.1 ng PA (blue) or 0.1 ng PB (magenta) presentation are shown as cumulative plots (A). For each sex pheromone stimulation, we calculated the slope of the plot using linear approximation and compared them between the initial 2-min period and the subsequent 2-min period. Solid lines and shaded areas indicate means and ± SEM, respectively. Total staying time during the initial 2-min period after the sex pheromone presentation was compared to that during the subsequent 2-min period using the Wilcoxon signed-rank test (B). (C, D) Typical locomotion patterns and staying times of no-treatment cockroaches to the mixture of PA and PB. No-treatment cockroaches which aroused by 0.05 ng of PB were stimulated by adding 0.05 ng of PA (PA+PB). The solid lines in upper panels in C show the movement of a selected cockroach during the first 2-min period of PB presentation (left panel) and the subsequent 2-min period of PA+PB presentation (right panel), and the red circle indicates the area close to the pheromone source (7 cm in diameter from the center of the petri dish). Changes of staying times from PB presentation (magenta) to PA+PB presentation (blue) are shown as cumulative plots and their slopes (C). Total staying time during the first 2-min period of PB presentation was compared that during the subsequent 2-min period of PA+PB presentation using the Wilcoxon signed-rank test (D).

**Table S1. Primers used in this study.**

**Table S2.** Sample numbers and statistical results.

(A) Sample numbers and results of statistical analysis used in RT-qPCR analysis and *in situ* hybridization denoted in Figure 3.

(B) Sample numbers and results of statistical analysis used in single sensillum recording analyses denoted in Figure 1, *SI Appendix* Supplementary Figure S3 and S4.

(B-1) PA responses of PA-SNs in no-treatment and RNAi cockroaches.

(A-2) PB responses of PB-SNs in no-treatment and RNAi cockroaches

(A-3) Response of PB-SNs to PA *PameOR1* RNAi cockroaches

(A-4) Response of PB-SNs to PB and/or PA *PameOR1* RNAi cockroaches

(B) Sample numbers and results of statistical analysis used in intracellular recordings from single L1-PNs denoted in Figure 3 and *SI Appendix* Supplementary Figure S5.

(B-1) sex pheromones responses of L1-PN in no-treatment and *PameOR2* RNAi cockroach

(B-2) Spontaneous firing frequency of L1-PN in no-treatment and RNAi cockroach

(C) Sample numbers and results of statistical analysis used in behavioral experiments denoted in Figure 4.

(E) Sample numbers and results of statistical analysis used in behavioral experiments denoted in Figure S6 and S7.

**Video S1. Behavioral responses of no-treatment and *PameOR1* RNAi cockroaches in response to 0.1 ng of PA.** Behavioral responses of three no-treatment (black dots) and three *PameOR1* RNAi (blue dots) cockroaches were simultaneously captured by an infrared camera during the 4 min period. The 0.1 ng of PA was placed into a petri dish located on the center of round arena. Solid line represents the locomotion activity of each cockroach during the 0.67-sec periods (equivalent to 20 frames) of before and after each of frames.

**Video S2. Behavioral responses of no-treatment and *PameOR2* RNAi cockroaches in response to 0.1 ng of PB.** Behavioral responses of three no-treatment (black dots) and three *PameOR2* RNAi (magenta dots) cockroaches were simultaneously captured by an infrared camera during the 4 min period. The 0.1 ng of PB was placed into a petri dish located on the center of round arena. Solid line represents the locomotion activity of each cockroach during the 0.67-sec periods (equivalent to 20 frames) of before and after each of frames.
