# Supplementary material for: Interactive parallel sex pheromone circuits that promote and suppress courtship behaviors in the cockroach
Source: PNAS Nexus. 2024 Apr 15;3(4):pgae162. doi: 10.1093/pnasnexus/pgae162 (PMC11058470; doi:10.1093/pnasnexus/pgae162)
Supplement: pgae162_Supplementary_Data [file pgae162_supplementary_data.zip › PNASNEXUS-PNASNEXUS-2023-01140-TR-s02.docx]

**
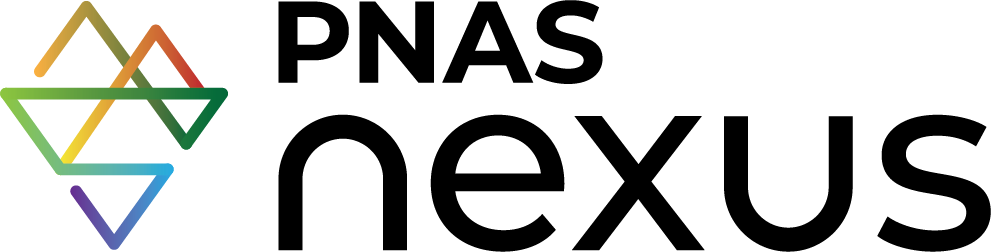
**

**Supplementary Information for**

**Interactive parallel sex pheromone circuits that promote and suppress courtship behaviors in the cockroach**

Kosuke Tateishi^1,2^, Takayuki Watanabe^3^, Mana Domae^4^, Atsushi Ugajin^5^, Hiroshi Nishino^4^, Hiroyuki Nakagawa^1^, Makoto Mizunami^4^, and Hidehiro Watanabe^1^*

1. Department of Earth System Science, Faculty of Science, Fukuoka University, Fukuoka 814-0180, Fukuoka, Japan

2. School of Biological and Environmental Sciences, Kwansei Gakuin University, Sanda 669-1330, Hyogo, Japan

3. Research Center for Integrative Evolutionary Science, The Graduate University for Advanced Studies Shonan Village, Hayama 240-0193, Kanagawa, Japan

4. Research Institute for Electronic Science, Hokkaido University, Sapporo 060-0812, Hokkaido, Japan

5. JT Biohistory Research Hall, Takatsuki 569-1125, Osaka, Japan

*Correspondence:

Dr. Hidehiro Watanabe

E-Mail: [nabehide@fukuoka-u.ac.jp](mailto:nabehide@fukuoka-u.ac.jp)

**This PDF file includes:**

Supplementary Methods

SI References

**Supplementary Methods**

**Molecular cloning of *PameORs***

We cloned four *OR* genes (*PameOR1*, *OR2*, *OR53*, and *OR62*) preferentially expressed in the male cockroach antennae reported by Chen et al. (1). First, we obtained partial cDNA sequences of the genes by conducting blastn searches in the NCBI SRA database (SRR3089536) with the nucleotide sequences of primers for RT-qPCR analysis in Chen et al. (1). Next, we performed RT-PCR to amplify cDNA fragments of the genes, and the 5’ and 3’ regions of the cDNAs were amplified by 5’ and 3’ rapid amplification of cDNA ends (RACE), respectively. Finally, the full-length open reading frame (ORF) of the genes was amplified using primers designed at the predicted 5´ and 3´-untranslated regions of the genes. All PCRs were performed using the Q5 High-Fidelity DNA polymerase (New England Biolabs, Tokyo, Japan). The 5’ and 3’ RACE were performed using the FirstChoice RLM-RACE kit (Ambion, Austin, TX, USA) according to Watanabe et al. (2). Primers used to amplify cDNAs containing full-length ORF are listed in *SI Appendix* Table S1. Amplified cDNA fragments were cloned into the pGEM-T Easy vector (Promega, WI, USA), and their nucleotide sequences were determined. The nucleotide sequences of the primers are listed in *SI Appendix* Table S1. The nucleotide sequence of the obtained cDNA was registered in GenBank (*PameOR1*; GenBank ID: LC781791*,* *PameOR2*; LC781792*, PameOR53*; LC781793*,* and *PameOR62*; LC781794).

**Quantitative reverse transcription PCR (RT-qPCR)**

The RT-qPCR analysis was conducted following the methods of our previous report (3) with modifications. RNA extraction and reverse transcription were carried out according to our previous report. The relative quantification of target genes was carried out using the KAPA SYBR Fast qPCR Kit (Kapa Biosystems, MA, USA) and the PCRmax Eco 48 Real-Time qPCR System (PCRmax, Staffordshire, UK). Gene expression levels were measured using the 2^-ΔΔCt^ method. The expression levels of *PameOR* mRNAs were normalized to that of *PameEf1α* (GenBank ID: LC657820). The nucleotide sequences of the primers are listed in *SI Appendix* Table S1.

**Preparation and injection of double-stranded RNA (dsRNA)**

The dsRNAs for *PameOR1, PameOR2, PameOR53,* and *PameOR62* were synthesized by T7 RNA polymerase-based *in vitro* transcription (3). A partial cDNA fragment of each target gene encoding to the C-terminal region including the sixth and seventh transmembrane helices was amplified with a pair of gene-specific primers with a T7 promoter sequence at the 5’ end, and was used as a template for *in vitro* transcription (The nucleotide sequences of the gene-specific primers used in the PCR are listed in *SI Appendix* Table S1). Template preparation and *in vitro* transcription were carried out according to our previous report (3).

We conducted the systemic RNAi as described in a previous study (3). 4-days-old adult males (RNAi*^Adult^*) or 7-days-old LI males (RNAi*^Nymph^*) were anesthetized on ice. A total of 4 μg of dsRNA of a target gene was manually injected into the dorsal region of the head capsule using a 27 G needle attached to a 10-μL glass syringe. The cockroaches injected with dsRNA were individually reared in isolated glass tubes and reared. In both RNAi*^Nymph^* and RNAi*^Adult^* groups, 11-days-old adult males were used for experiments. The control group consisted of 11-days-old virgin males with no treatment or those injected with 4μg of *β-lactamase* dsRNA (Control). In our previous article conducting the *PameORco* RNAi (3), we observed a significant decrease in *PameORco* expression levels in RNAi*^Nymph^* and RNAi*^Adult^* groups. However, the RNAi efficiency in the RNAi*^Nymph^* cockroach is stronger than that in the RNAi*^Adult^* cockroach.

***In situ* hybridization of *PameOR2* and *PameORco***

We performed *in situ* hybridization following the protocols described in the previous study with some modifications (4). Digoxigenin (DIG)-labeled cRNA probes for *PameOR2* and *PameORco* were synthesized by *in vitro* transcription. Template DNA fragments were amplified by PCR with a gene-specific primer and a gene-specific primer containing the T7 promoter sequence at the 5’ end (*SI Appendix* Table S1). Then, DIG-labeled sense and anti-sense probes were synthesized using the DIG RNA Labeling Mix (Roche) and the HiScribe T7 High Yield RNA Synthesis Kit (New England Biolabs).

To perform *in situ* hybridization, the antennae from one-day-old adult male cockroaches were dissected and divided into several fragments. The antennal fragments were then embedded in the Tissue-Tek optimal cutting temperature compound (Sakura, Tokyo, Japan), frozen in liquid nitrogen, and sliced along the longitudinal axis at 14 μm thickness using a Cryostat (CM1850, Leica). The sections were mounted onto glass slides with a hydrophilic coating (Platinum micro slide glass, Matsunami, Osaka, Japan). Sections were air-dried and stored at -80°C until use for hybridization. After fixation of sections in 10% formaldehyde neutral buffer (pH 7.0, Nakalai-Tesque), specimens were permeabilized with 0.3% Triton X-100 in phosphate-buffered saline (PBS). The antennal sections were incubated with either sense or anti-sense DIG-labeled cRNA probes at 52°C overnight in hybridization buffer [50% formamide, 5×SSC (0.75 M NaCl, 0.075 M sodium citrate, pH 7.0), 100 mg/ml of yeast tRNA (Roche), 0.1% Tween 20, and 50 mg/ml heparin]. Concentrations of the cRNA probes were adjusted so that antisense and sense probes have equivalent titers. After then, sections were washed for 30 min at 52°C in 50% formamide in 5×SSC, and then for 30 min at 52°C with 50% formamide in 4×SSC. Sections were further equilibrated twice with Tris Buffer (10 mM Tris-HCl (pH 8.0), 500 mM NaCl) at room temperature each for 5 min. After treatment with RNase A (20 µg/ml in Tris Buffer) at 37°C for 30 min, sections were washed with 50% formamide in 1×SSC for 30 min at 52°C. After equilibration with maleic acid buffer (0.1 M maleic acid, 0.15 M NaCl, pH 7.5), sections were blocked in 1% blocking reagent (Roche) in maleic acid buffer, followed by reaction overnight with 1:800 diluted alkaline phosphatase-conjugated anti-Dig antibody (Roche) at 4°C overnight. Sections were washed with maleic acid buffer again and then equilibrated with 100 mM Tris-HCl (pH 9.5), 100 mM NaCl, 50 mM MgCl_2_ (reaction buffer). The signal was visualized using 5-bromo-4-chloro-3-indoyl phosphate and nitro blue tetrazolium.

**Fluorescence *in situ* Hybridization (FISH) of *PameORs***

We performed fluorescence *in situ* hybridization (FISH) following the protocols described in the previous study with some modifications (5, 6). Riboprobes were prepared by *in vitro* transcription using PCR products as a template (*SI Appendix* Table S1). Digoxigenin (DIG)-, fluorescein (FLU)- and 2,4-dinitrophenyl (DNP)-labeled riboprobes were synthesized using RNA labeling kits (Roche, Basel, Switzerland). Synthesized products were purified by LiCl precipitation.

The antennae from four-days-old adult male cockroaches were dissected and divided into several fragments. The antennal fragments were then embedded in the Tissue-Tek optimal cutting temperature compound (Sakura, Tokyo, Japan), frozen in liquid nitrogen, and sliced along the longitudinal axis at 10 μm thickness using a Cryostat (OT/FAS/EC/MR/Z, Bright Instrument Company, UK) set at −18°C. Sections were collected on APS-coated microscope slides (Matsunami Glass Ind, Osaka, Japan). After overnight airdrying, sections were fixed in 4% paraformaldehyde in 0.1M phosphate buffer (PB) overnight at 4°C, treated with 12.5µg/ml proteinase K (29442-14, Nacalai, Kyoto, Japan) for 15 min and then with 0.2 M HCl for 10 min, followed by acetylation solution [0.25% acetic anhydride, 0.1 M Triethanolamine hydrochloride (T1502, Sigma-Aldrich, MO, USA)] for 10 min at room temperature. Slides were rinsed with PB between each step. After dehydration through a series of ethanol solutions (70, 80, 90, and 100%), sections were hybridized with the riboprobes overnight at 60 ◦C. The riboprobes were diluted in hybridization buffer (50 % formamide, 10 mM Tris-HCl (pH 7.6), 200 µg/ml yeast tRNA (15401-011, Thermo Fisher Scientific, MA, USA), 50 µg/ml heparin, 1× Denhardt’s solution, 100 mg/ml sodium dextran sulfate, 0.6 M NaCl, 0.25% SDS, 1 mM EDTA) at a concentration of 1 µl/ml, heat-denatured at 85°C for 5 min, and then added to each slide. A strip of Parafilm (Bemis, IL, USA) was placed on top and slides were incubated in a box moisturized with 50 % formamide at 60 ◦C overnight. After hybridization, slides were washed in a wash solution [50 % formamide, 2× standard sodium citrate (SSC)] at 60°C for 30 min, treated with 6.25 µg/ml RNase A (30142-04, Nacalai) in Tris-NaCl-EDTA buffer (10mM Tris-HCl (pH 7.6), 1 mM EDTA, 0.5 M NaCl) at 37°C for 30 min, and washed at 60°C in 2× SSC for 20 min and twice in 0.2× SSC for 20 min. Slides were then blocked with TrisNaCl-Blocking (TNB) buffer [0.1 M Tris-HCl (pH 7.6), 0.15 M NaCl, 5 mg/ml blocking reagent (FP1020, PerkinElmer)] for 1 h at room temperature.

Signals were detected immunocytochemically by a combination of peroxidase (POD)-conjugated antibody and yramide signal amplification (TSA) system. After blocking, slides were incubated with anti-DIG-POD (11207733910, Roche) diluted in TNB buffer (1:500) one or two overnight at 4 ºC , washed three times in Tris-NaCl-Tween20 (TNT) buffer [0.1 M Tris-HCl (pH 7.6), 0.15 M NaCl, 0.05% Tween20] for 5min, further rinsed three times in borate buffer [0.1 M H3BO3 (pH 8.5), 0.1% Tween20], and incubated with TSA reaction solution (20 mg/ml sodium dextran sulfate, 0.3 mg/ml 4-iodophenol, 0.003% H_2_O_2_ in borate buffer) containing TSA Plus Cy3 Reagent (1:200; TS-000202, Akoya Biosciences, MA, USA) for 30 min at room temperature without shaking. For multicolor staining, after the first TSA reaction, slides were washed in 0.1% Tween20 containing PB (PBT) and incubated in 3% H_2_O_2_ containing PBT for 10min at room temperature to inactivate POD, followed by three times washing in TNT buffer. Slides were then incubated with another POD conjugated antibody, and we performed the abovementioned signal detection process repeatedly. FLU- and DNP-labeled riboprobes were detected in combination with 5-(and-6) carboxyfluorescein tyramide (kindly provided by Dr. Yasuko Akiyama-Oda) and TSA Plus Cy5 Reagent (TS-000203, Akoya Biosciences), respectively. Fluorescent images were captured using a confocal scanning microscope (LSM980, Zeiss, Germany).

**Intracellular recording and staining of L1-PN**

The procedures for the intracellular recording and staining from L1-PN were described in detail previously (7). A glass microelectrode filled with 8% Lucifer Yellow (Sigma-Aldrich) in 1 M LiCl_2_ was adjusted the input resistances as 30-50 MΩ. The cockroach was anesthetized by carbon dioxide and then fixed onto a handmade acrylic chamber using wax. The head was positioned within a shallow chamber and fixed with low-melting wax. To expose the brain surface, a small rectangular window was cut in the head cuticle between the compound eyes, followed by the removal of muscles and trachea. The brain was immersed in cockroach saline to establish an electrically connection with an indifferent silver rod (300 µm in diameter), which was inserted into a cavity between the circumesophageal connectives. For intracellular recording and subsequent staining, the electrode was inserted into the axon just below the point where two calyces meet (arrowheads in Fig. 3A). Synaptic potentials elicited by pre-synaptic neurons could not be measured due to the insertion site being distant from the dendrites. The electrical signals were amplified by a DC amplifier (MEZ-8301, Nihon Kohden, Japan), displayed on an oscilloscope, and then digitized. L1-PNs were readily identifiable based on their spontaneous activities and responsiveness to PB. The spontaneous spike frequency of L1-PN was calculated for a period of 1 s before the onset of sex pheromone stimulus. We presented 1 ng of PB, or PA, or a mixture of 1 ng of PA and 1 ng of PB (PA+PB) to antennae with a duration of 0.3 s. Each sex pheromone stimulus was randomly presented 3-7 times, with intervals of > 30 s.

After recording the olfactory responses, the L1-PN was filled with Lucifer Yellow by injecting a hyperpolarizing current. After staining, the electrode was removed from the brain, and the head capsule was fixed on a wax plate. Anterograde staining of antennal afferents was then conducted following previously described protocols (7, 8). The double-stained brain was fixed, dehydrated, cleared, and then observed by Laser scanning microscope (LSM-980; Carl Zeiss, Jena, Germany). The contrast and brightness of all images were adjusted using Adobe Photoshop CS3 and Illustrator CS3.

Spikes of L1-PNs are sorted and counted using the spike sorting function of Spike 2 ver. 8.08 (CED, Cambridge, UK). To evaluate the temporal activity patterns evoked by sex pheromones, we generate raster plots and peri-stimulus time histograms with 20 ms bins. All recorded L1-PNs in no-treatment cockroaches exhibits excitatory response to PB, albeit with slightly varying response latencies among individuals. Consequently, we reorganized the spike arrays elicited by PB, PA and PA+PB stimuli for each individual, utilizing the onset of PB response as a reference point. The onset of PB response was identified as the spike exceeding an instantaneous spike frequency of >150Hz. Statistical analysis was performed using one-way ANOVA and post-hoc Tukey-Kramer test. Detailed results of statistical analysis were summarized in *SI Appendix* Table S2.

**SI References**

1. Y. Chen, M. He, Z.Q. Li, , Y.N. Zhang, P. He, Identification and tissue expression profile of genes from three chemoreceptor families in an urban pest, *Periplaneta americana*. *Sci. Rep.* **6**, 27495 (2016).

2. T. Watanabe, A. Ugajin, H. Aonuma, Immediate-early promoter-driven transgenic reporter system for neuroethological research in a hemimetabolous insect. *eNeuro* **5**. 10.1523/ENEURO.0061-18.2018 (2018).

3. K. Tateishi, T. Watanabe, H. Nishino, M. Mizunami, H. Watanabe, Silencing the odorant receptor co-receptor impairs olfactory reception in a sensillum-specific manner in the cockroach. *iScience* **25**, 104272 (2022).

4. S. Fukunaga, R. Matsuo, S. Hoshino, Y. Kirino, Novel kruppel-like factor is induced by neuronal activity and by sensory input in the central nervous system of the terrestrial slug Limax valentianus. *J Neurobiol* **66**, 169-181 (2006).

5. A.-O. Yasuko, H. Oda, Multi-color FISH facilitates analysis of cell-type diversification and developemental gene regulation in the Parasteatoda spider embryo. *Develop. Growth Differ.,* 58. 215-224 (2016)

6. A. Ugajin, K. Ozaki, Coexpression of Three Odorant-Binding Protein Genes in the Foreleg Gustatory Sensilla of Swallowtail Butterfly Visualized by Multicolor FISH Analysis. Front. *Insect Sci.*, 1:696179, (2021)

7. H. Nishino *et al.*, Spatial Receptive Fields for Odor Localization. *Curr Biol* **28**, 600-608 e603 (2018).

8. H. Watanabe, H. Nishino, M. Nishikawa, M. Mizunami, F. Yokohari, Complete mapping of glomeruli based on sensory nerve branching pattern in the primary olfactory center of the cockroach Periplaneta americana. *J Comp Neurol* **518**, 3907-3930 (2010).
